# Occurrence of anticancer drugs in the aquatic environment: a systematic review

**DOI:** 10.1007/s11356-019-07045-2

**Published:** 2019-12-12

**Authors:** Carla Nassour, Stephen J. Barton, Shereen Nabhani-Gebara, Yolande Saab, James Barker

**Affiliations:** 1 School of Life Sciences, Pharmacy and Chemistry, Kingston University, Penrhyn Road, Kingston Upon Thames, KT1 2EE UK; 2 School of Pharmacy, Lebanese American University, Beirut, Lebanon

**Keywords:** Systematic review, Pharmaceuticals, Anticancer drugs, Sampling, Analytical techniques, Aquatic environment

## Abstract

Water contamination with pharmaceutical products is a well-studied problem. Numerous studies have demonstrated the presence of anticancer drugs in different water resources that failed to be eliminated by conventional wastewater treatment plants. The purpose of this report was to conduct a systematic review of anticancer drugs in the aquatic environment. The methodology adopted was carried out in compliance with the PRISMA guidelines. From the 75 studies that met the specific requirements for inclusion, data extracted showed that the most common anticancer drugs studied are cyclophosphamide, tamoxifen, ifosfamide and methotrexate with concentrations measured ranging between 0.01 and 86,200 ng/L. There was significant variation in the methodologies employed due to lack of available guidelines to address sampling techniques, seasonal variability and analytical strategy. The most routinely used technique for quantitative determination was found to be solid-phase extraction followed by LC-MS analysis. The lowest reported recovery percentage was 11%, and the highest limit of detection was 1700 ng/L. This indicated the inadequacy of some methods to analyse anticancer drugs and the failure to obtain reliable results. The significant heterogeneity within methodologies made it difficult to compare results and draw conclusions, nevertheless, this study aids in the extrapolation of proposed recommendations to guide future studies and reviews.

**Electronic supplementary material:**

The online version of this article (10.1007/s11356-019-07045-2) contains supplementary material, which is available to authorized users.

**Figure:**
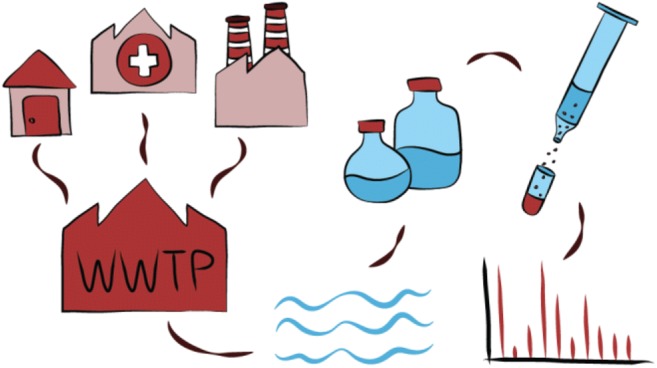
Graphical abstract

## Introduction

Pharmaceuticals are currently identified as pseudo-persistent compounds seeing that they are constantly released into the aquatic environment (Ebele et al. 2017). In fact, studies conducted as early as the 1970s in the USA revealed the presence of numerous pharmaceuticals in wastewater. Since then, many improvements in analytical methodologies have facilitated the detection of very low concentrations of pharmaceuticals in surface water, wastewater, groundwater and drinking water (World Health Organization 2012). According to a critical review conducted by Stephen R. Hughes et al. in 2013, antibiotics, antiepileptics, cardiovascular drugs and painkillers are the most studied compounds possibly due to frequent consumption worldwide. In contrast, other potentially toxic therapeutic classes such as anticancer drugs have not received much attention (Hughes et al. 2013).

Anticancer drugs are categorised by the Anatomical Therapeutic Chemical (ATC) classification system into two groups according to their therapeutic, pharmacological and chemical characteristics: antineoplastic agents (L01) and endocrine therapy (L02) (Besse et al. 2012). Antineoplastic drugs are classified into five groups: L01A alkylating agents, L01B antimetabolites, L01C plant alkaloids and other natural products, L01D cytotoxic antibiotics and related substances and L01X other antineoplastic agents. Meanwhile, in endocrine therapy, hormones (L02A), anti-hormones and related agents (L02B) are utilised (Besse et al. 2012; Xie 2012).

The production and consumption of anticancer drugs are on the rise corresponding to the increased incidence of cancer worldwide (Ferrando-Climent et al. 2014). Considering their significant effect on human cells and hormone systems, there is concern about the environmental risk of anticancer drugs (Besse et al. 2012). For this reason, several studies have investigated the acute and chronic effects of anticancer drugs in the aquatic environment: for acute toxicity, concentrations that are likely to cause negative effects on aquatic organisms are found to be greater than the concentrations detected in water, except in a case of a spill (Fent et al. 2006). That means that acute effects are very improbable. Despite the fact that there are not many studies on chronic toxicity, some tests showed that the concentrations detected in water were higher than the EC_50_ (half maximal effective concentration) which implies that trace concentrations of anticancer drugs in the water may provoke adverse effects on the long-term or/and when they are present in a mixture (Xie 2012; Booker et al. 2014; Franquet-Griell et al. 2017; Santos et al. 2017).

Previous studies and reviews examining the occurrence of anticancer drugs in environmental samples mainly focused on the analytical techniques applied to detect these compounds and their physico-chemical properties that affect their presence in the aquatic environment (Kosjek and Heath 2011; Nussbaumer et al. 2011; Gómez-Canela et al. 2013; Santana-Viera et al. 2016). Therefore, the aim of this report was to conduct an exhaustive systematic review of all available studies that have investigated the presence of anticancer drugs in the aquatic environment to date, in compliance with the PRISMA (Preferred Reporting Items for Systematic Reviews and Meta-Analyses) checklist. In addition, the corresponding sampling strategies and methodologies adopted were discussed in an effort to assess the quality and validity of the included studies.

Throughout this review, surface water, groundwater, wastewater and drinking water will be referred to using the term “aquatic environment”.

## Selection process

A preliminary literature search was conducted on different databases and PROSPERO to check if the subject is qualified for a review by the number and type of publications found and to ensure that a similar review was not already published. Following the scoping searches, a review protocol was made and registered in PROSPERO (number of registration: CRD42018100457).

In order to develop the inclusion criteria, a PICO table, shown in Table 1, was generated including the Population, Intervention, Comparator, Outcome and the Study Design. All original studies written in English, published or not (Grey literature), that assess at least one anticancer drug were included in this review with no restriction on the year of the study. Also, all types of findings were included (positive and negative) in a way to reduce publication bias and to have a representative search of all of the studies conducted. Editorials, newspaper articles, reviews or other forms of popular media were excluded. In addition, studies that treat other matrices than water or analyse the elimination or the stability of anticancer drugs were rejected.

**Table 1:** Eligibility criteria (PICO table)

Eligibility criteria	
Population	All types of anticancer drugs studied in the aquatic environment
Intervention	All types of sampling strategies, extraction and analytical techniques
Comparator	The stated interventions will be compared with each other
Outcome	Any positive or negative results will be included
Study design	Original studies; studies that analyse at least one anticancer drug

In the interest of identifying as many significant papers as possible, published or not, a review was conducted through a search on PubMed and OpenGrey on June 25, 2018. Simultaneously, the same research was applied to the database ScienceDirect to verify that no other significant publications were available. The searched terms were combined using Boolean operators and wildcards: (Pharmaceutical* OR Anticancer$ OR Antineoplastic$ OR Cytotoxic$ OR Drug$ OR Organic OR Cytostatic$ OR Residue$ OR PhA OR PhAC) AND (Detection OR Extraction OR Occurrence OR Analysis OR Determination OR Incidence OR Assessment) AND (*Water* OR Wastewater OR Aquatic OR River OR Sewage) AND (SPE OR Solid-phase extraction OR LC OR Chromatography OR Liquid Chromatography).

The initial search on PubMed covered 23,766 publications from 1964 until the present, while OpenGrey reported only 128 studies from 1993 until 2013. It was refined by: Languages = English (PubMed and OpenGrey) and Sort by = Best Match (PubMed). Therefore, the study was narrowed to 5575 publications from PubMed and 46 from OpenGrey.

Two stages were required to apply the inclusion criteria as reported by PRISMA. Firstly, articles were selected according to the title and abstract. All studies that do not appear to meet all criteria were excluded, whereas, if it is unclear that all the inclusion criteria were met, the publication was included for further study. After the screening stage, the full texts of the remaining publications were obtained to select the articles that substantially met the eligibility criteria. All the rejected references were reported with the reason of exclusion in the PRISMA diagram. Furthermore, bibliographies of excluded and included studies were examined to determine more pertinent publications that might be missed during the research. The screening and selection steps were accomplished by two reviewers independently. The relevance of each study was assessed according to the inclusion criteria stated in Table 1, and any disagreement was resolved by consensus.

The next task was to examine the quality of each of these studies, which meant the evaluation of the validity, reliability and generalisability of the results. To accomplish this, the number of anticancer drugs tested was considered as the sampling strategy, if the number of samples was representative, if the methods of extraction and analysis were optimised and validated and, finally, how the results were reported.

In this review, two types of data were extracted: the descriptive data, which includes the study, the year, the country, the compound tested, the strategy of sampling, the number of samples and the extraction, separation and detection techniques, and the analytical data, which covers the outcomes (number of detections and no detections and the concentration of each compound found in the water cycle). All data were extracted by one reviewer and checked for accuracy by a second reviewer.

**Fig. 1:**
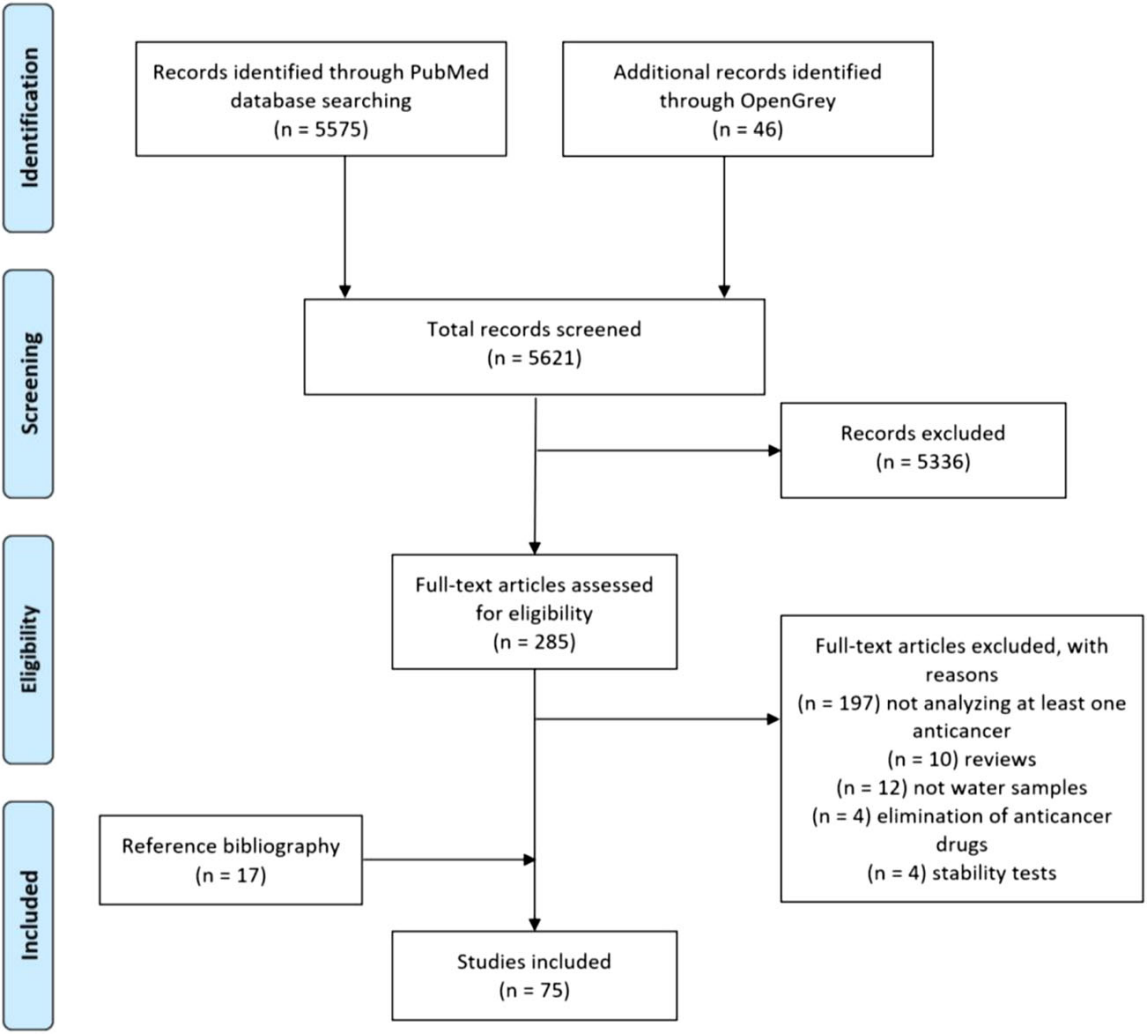
Identification of included studies in the systematic review

Following the refined search on PubMed and OpenGrey, 5621 citations were identified in total to be screened and selected for inclusion. Titles and abstracts were assessed, determining 285 publications (Stage 1 screening), by which their full texts were obtained. Afterwards, the inclusion criteria were applied to the full text (Stage 2 selection), resulting in 58 citations being retained, while 197 studies were excluded for not analysing at least 1 anticancer drug; 10 reviews were eliminated; 12 others did not assess water samples; 4 articles which studied the elimination of anticancer drugs and 4 which examined their stability were rejected. In addition, 17 publications were included from bibliography searches. The study selection process is shown in the PRISMA diagram (Fig. 1).

## Data extraction

About 75 studies met the inclusion criteria and were subsequently reviewed in order to extract the information needed to complete the review. The data were arranged in Table S1 (Supplementary Information 1) and included the publication reference, the year the study was conducted, the country where the samples were taken, the anticancer drug tested, the type and number of water samples examined, the methods of the analysis and the resulting concentrations reported in ng/L to facilitate the comparison between different studies.

The studies were analysed considering the following parameters: geographical representation, choice of anticancer studied, sampling strategy and analytical techniques.

## Geographical representation

From the reviewed manuscripts, the earliest study was in 1996 (Steger-Hartmann et al. 1996). The majority of the studies were conducted in Spain (24 studies) and the UK (13 studies). Therefore, fewer investigations have been conducted in other countries. Thus, the global scope of the problem cannot be assessed. Moreover, studies conducted in each country focused on specific areas, limiting representation.

## Frequently studied compounds

**Fig. 2:**
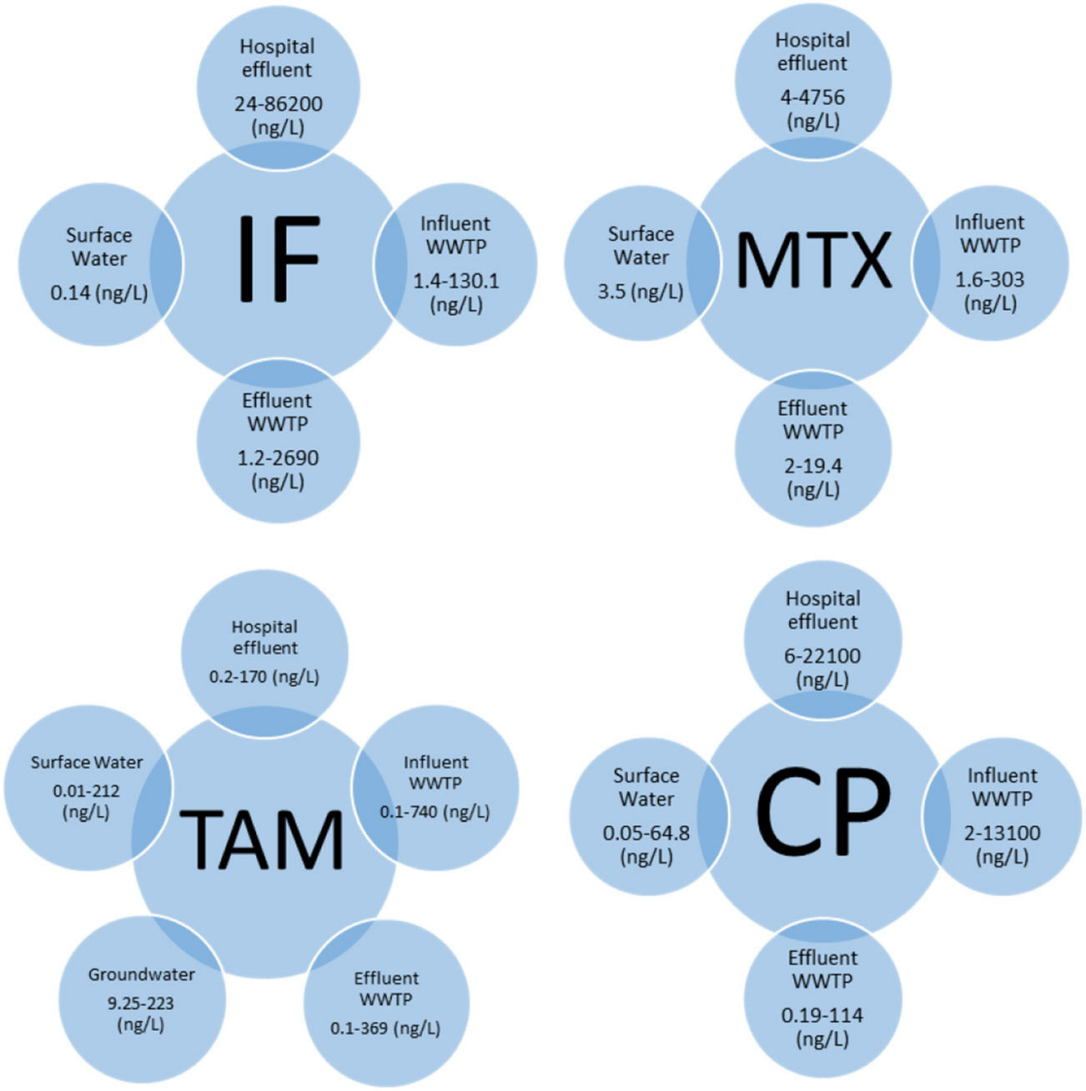
Summary of the findings for the four most frequently studied anticancer drugs

Among the 75 included publications, the 4 most studied anticancer agents were cyclophosphamide (39), tamoxifen (30), ifosfamide (29) and methotrexate (17), possibly because they are the most commonly used anticancer drugs in the corresponding countries (Tauxe-Wuersch et al. 2006; Yin et al. 2010b). Concentrations of the alkylating agents, cyclophosphamide and ifosfamide, ranged from 0.05 to 22,100 ng/L and from 0.14 to 86,200 ng/L in 24 and 16 studies, respectively. The antimetabolite, methotrexate, was detected at concentrations between 1.6 and 4756 ng/L in 13 studies out of 17. Finally, the hormonal agent, tamoxifen, was identified in 19 articles with concentrations varying between 0.01 and 740 ng/L (Fig. 2).

Additionally, active metabolites of other anticancer drugs were studied and detected in water samples. These include endoxifen (OH-D-TAM), (Z)-4-hydroxytamoxifen (OH-TAM), hydroxymethotrexate (OH-MET), hydroxypaclitaxel (OH-PAC), 1-β-d-arabinofuranoside (AraU) and 2’,2’-difluorodeoxyuridine (dFdU) (Santana-Viera et al. 2016) (Table S1).

The included studies revealed that diverse methodological approaches were adopted to measure anticancer drugs in the aquatic environment. This could explain the significant variation in anticancer concentrations detected in these studies. Moreover, sample sources also seemed to affect the results obtained. Consequently, studies demonstrated that the detection of anticancer compounds can be affected by several external factors, hence limiting the ability to compare different studies and infer conclusions.

The diversity in employed methodologies was due to several variability factors that will be discussed below.

## Variability factors

### Sample source

**Fig. 3:**
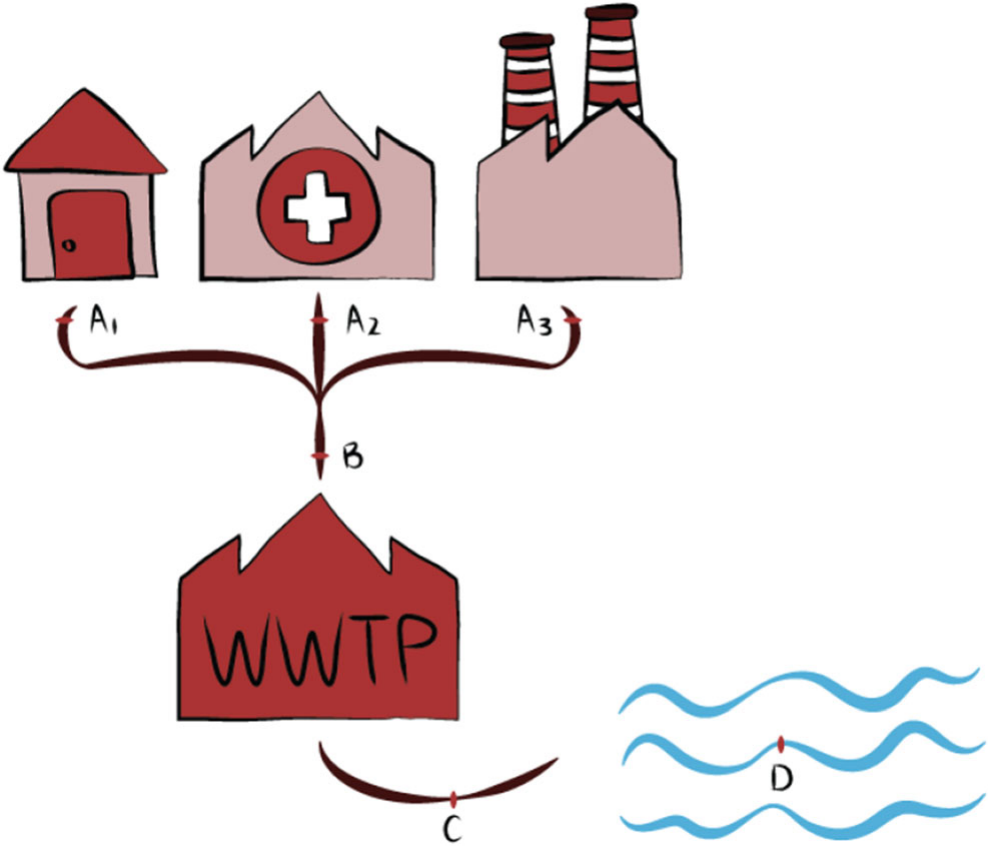
Sample sources ((A) raw wastewater from (1) households, (2) hospitals or (3) industries; (B) influent of wastewater treatment plants or untreated wastewater; (C) effluent of wastewater treatment plants or treated wastewater; (D) surface water, sea, etc.)

Sample sources ranged mainly from wastewater from hospitals, influent and effluent of wastewater treatment plants (WWTP) and surface water (Fig. 3). Results showed that the concentrations of anticancer drugs can fluctuate if the sampling was near the hospital or in rural or urban areas (Iglesias et al. 2014). Also, results changed according to the size of the hospital, the number of patients treated, the presence of specific departments (if it only treats cancer or not), the drugs used for cancer therapy, the consumption rate and the day of the sampling (some studies demonstrated a difference between weekday samples and weekend samples due to the reduced therapy on weekends) (Steger-Hartmann et al. 1996; Verlicchi et al. 2012; Rabii et al. 2014; Česen et al. 2015, 2016). In Weissbrodt et al.’s study, 5-fluorouracil was assumed to be highly detected in the hospital wastewater since it was the most consumed anticancer drug in Cantonal hospital and especially on Wednesday when it was administered the most (Weissbrodt et al. 2009).

In general, concentrations are higher in hospitals’ wastewater or wastewater influents than wastewater treatment plant effluents and surface water (mainly from rivers) taking into consideration the dilution factor, the elimination or inactivation by WWTPs or the adsorption of some compounds onto sludge. Numerous studies demonstrated this matter such as the survey conducted by Roberts and Thomas on tamoxifen. Concentrations of this compound ranged between 376 and 740 ng/L in pretreated wastewater, decreased to 146–369 ng/L in treated wastewater and to 27–212 ng/L in surface water (Roberts and Thomas 2006). This can be explained by the dilution factor, the characteristics of the sampling location (*e.g.* width, depth, flow rate of the water), the type of wastewater treatment performed in each plant and its elimination efficiency (López-Serna et al. 2012; Osorio et al. 2012; Idder et al. 2013). For instance, a typical example of elimination studies on some anticancer drugs revealed that cyclophosphamide, ifosfamide, vinblastine, vincristine, etoposide, doxorubicin, epirubicin, daunorubicin and cisplatin are not biodegradable, whereas 5-fluorouracil showed negative and positive results. It also seemed that degradation was not as prevalent in the presence of other anticancer compounds, when within a mixture (Kosjek and Heath 2011). Moreover, cyclophosphamide, ifosfamide and 5-fluorouracil appeared to not adsorb onto sludge or sediments and did not undergo direct photolysis demonstrated by their high persistence in the environment (Kiffmeyer et al. 1998; Buerge et al. 2006; Kosjek and Heath 2011).

### Sampling strategy

The different sampling methods for the four most studied compounds are reported in Table S2 (Supplementary Information 1). It is apparent that no standardised sampling strategy has been adopted in all of the included studies seeing that the number of samples ranged from 1 to 105. In consequence, the focus of most of the papers was on the development of the analytical methods and their validation using environmental samples rather than obtaining a representative number of samples that can accurately illustrate the variable levels of anticancer drugs persisting in the aquatic environment. Additionally, the majority of the samples were collected as 24 h composite samples or as grab samples. In Busetti et al.’s study, comparison between these two types of sampling was carried out. Results demonstrated that composite samples were more representative and showed less variations in the concentrations, whereas concentrations obtained from grab samples appeared to be more irregular and time-dependent (Busetti et al. 2009). In some cases, obtaining composite samples could be very challenging since hospitals or treatment plants need to authorise it (Yin et al. 2010a). In a critical review completed by Ort et al. (2010), the authors recommended to provide more details about the sampling strategy in order to prevent the uncertainty of the results obtained and the misleading interpretations taking into consideration several factors such as the goal of the study, the sampling mode and frequency and location, etc. For example, for analytical methods development or for qualitative studies, grab sampling can be sufficient, whereas for screening studies, composite samples are more recommended.

### Season variability

In addition, variation can be observed depending on the period of the sampling. Some researchers collected their samples from rivers, WWTP and hospitals effluent in hot weather seasons to prevent dilution by rainfalls and high water flows (Ashton et al. 2004; Verlicchi et al. 2012). In contrast, many studies demonstrated that greater concentrations are detected in winter (in river samples and wastewater influent and effluent) due to the low temperatures and reduced radiation from the sun, which limits the biodegradation of pharmaceuticals (Osorio et al. 2012; Kot-Wasik et al. 2016). However, one study showed that the concentration of cyclophosphamide was not affected by the change of seasons (Rabii et al. 2014). Nevertheless, the results of this study could not be confirmed since the WWTP influent and effluent samples were not collected under the same conditions (different consumption rate of this drug on these sampling days and/or collection of samples at different time which can affect the concentration of the anticancer drug in the wastewater (e.g. morning urine)).

### Target compound

The presence or the absence of an anticancer drug in the aquatic environment is affected by the form of its elimination from the body: parent compound or metabolised form (Yin et al. 2010a; Gómez-Canela et al. 2014) and the corresponding stability in the water when excreted (Garcia-Ac et al. 2009a; Negreira et al. 2014a). Studies conducted by Gomez-Canela et al. and Yin et al. in 2012 and 2009, respectively, observed high excretion rates for some anticancer agents, e.g. gemcitabine, 92–98% (urine); megestrol, 66% (urine) and 20% (faeces); methotrexate, 60–95% (urine); etoposide, 44–60% (urine) and > 16% (faeces); cyclophosphamide, 5–25% (urine) and 31–66% (faeces); ifosfamide, 14–50% (urine); and tamoxifen, 9–13% (urine) and 26–65% (faeces), among others (Yin et al. 2010a; Gómez-Canela et al. 2013). This can also mislead the interpretation of the results obtained since the absence of one parent compound can be explained by the presence of its metabolite.

### Sample storage

Determination of suitable conditions to preserve water samples was essential considering that concentrations of anticancer drugs in environmental samples can easily change and be underestimated. Also, after the sampling procedure, water cannot always be analysed in the same day. In consequence, stability tests were carried out for numerous anticancer drugs in which variations of time, temperature and pH were made to determine the ideal storage conditions of wastewater samples. Results showed that some anticancer drugs are hydrophilic and may persist more in the aquatic environment e.g. ifosfamide, cyclophosphamide and 5-fluorouracil, among others. Additionally, preservation of water samples at -20 °C showed better recoveries than 4 °C and 25°C for most of the compounds where degradation started to increase gradually with the temperature. Besides, acidification of samples increased the stability of some compounds like 5-fluorouracil, vinorelbine, erlotinib and capacetabine, but may degrade others such as ifosfamide. For this reason, it is recommended to freeze the samples directly after collection at -20 °C and analyse them as soon as possible (Ferrando-Climent et al. 2013; Negreira et al. 2014a, b).

## Extraction and determination techniques

### Extraction

Pretreatment of environmental samples is an essential step seeing the complexity of the matrix (indicating the constituents of the sample other than the analyte of interest) and the low concentrations of the compounds resided in the aquatic environment. The purpose of this step is to concentrate the analytes in question while trying to eliminate probable interferences (Chapuis et al. 2005). The majority of the included studies used solid-phase extraction (SPE) for the sample preparation consisting of the extraction and the preconcentration of anticancer drugs (Table S3). Solid-phase extraction can be operated in offline and online modes. In studies that adopted offline SPE, numerous cartridges were used demonstrating different extraction efficiencies for anticancer compounds. This indicates that the choice of the right type of cartridges depends on the nature of the sorbent and its ability to retain the anticancer drug of interest. Oasis HLB cartridges has offered a good hydrophilic and lipophilic balance allowing the retention of a large range of compounds. For this reason, Oasis HLB cartridges were utilised in the majority of the studies, since better recoveries were obtained for several anticancer drugs with different physico-chemical characteristics (Ferrando-Climent et al. 2013). For anthracycline compounds, C8 columns appeared to be the most adequate especially for epirubicin, doxorubicin and daunorubicin (Mahnik et al. 2007). However, in multi-residue methods, Strata-X cartridges have not provided good recovery percentages for tamoxifen (Nebot et al. 2007). The cartridges employed in online SPE were Strata-X, HySphere Resin GP, PLRP-s and Hypersil GOLD PFP. Strata-X cartridges were mostly utilised, providing good recoveries for cyclophosphamide, methotrexate and ifosfamide (Garcia-Ac et al. 2009a, 2009b, 2011; Idder et al. 2013). Analysis performed automatically showed higher recovery rates and improved sensitivity for the detection of anticancer agents. In fact, online SPE proved to be more convenient since it is a faster method, demands a smaller volume of sample, decreases sample loss throughout the manipulation and reduces impurity contamination (Kosjek and Heath 2011; Santana-Viera et al. 2016). Along with selecting the right cartridges, several parameters require optimisation for a higher recovery, including the elution solution. In general, polar solvents were used to elute the compounds of interest such as acetone, methanol and acetonitrile. Besides, gradient elution was also applied using online SPE for better extraction efficiencies (Garcia-Ac et al. 2009a, b, 2011).

### Analytical techniques

HPLC-MS^2^ was mostly used for the separation and detection of anticancer drugs (Table S3). Studies revealed that this technique was more suitable and offered higher sensitivity than GC-MS, avoiding a derivatization step, since most of these compounds are susceptible to degradation at high temperatures and have low volatility and high molecular weight (Kosjek and Heath 2011). Also, electrospray ionisation (ESI) seemed to be the most utilised ionisation technique applied among others. For instance, a comparison of different methods of ionisation was made by Garcia-Ac et al. to finally conclude that ESI has higher sensitivity and selectivity (Garcia-Ac et al. 2011). However, in some studies using GC-MS, a lower limit of detection was achieved for some compounds such as 5-Fluorouracil (5-FU) (Kosjek et al. 2013)*.* Additionally, other analytical techniques were employed with good results for specific compounds. For example, capillary electrophoresis with diode array detection was used for 5-FU determination enhancing the separation rate and selectivity with a lesser sample volume. In addition, it is faster than HPLC and inexpensive (Mahnik et al. 2004, 2007). Inductively coupled plasma mass spectrometry was used for the detection of platinum-based anticancer drugs such as cisplatin (Vidmar et al. 2015; Isidori et al. 2016).

### Validation parameters

The majority of the studies included in this review developed and validated their own methods. Validation was achieved using reference standards of each compound prepared in suitable solvents, mainly methanol or a mixture of water and methanol (Nebot et al. 2007; Busetti et al. 2009; Garcia-Ac et al. 2011). The conducted calibration plot over the analytical working range achieved linearity with a correlation coefficient (R^2^) between 0.97 and 0.99. The precision, expressed as a percentage of the relative standard deviation, should be smaller than 20% for water analysis (Camacho-Muñoz et al. 2016), whereas it went from 0.1 reaching 80%, which means that some of the given results are not reliable.

Overall, in the 75 publications examined, the accuracy of the method expressed as recovery percentage, ranged from 11 to 167% and limits of detection varied from 0.003 to 1700 ng/L. Low recovery percentages and high LODs in some studies indicated that the adopted methods were inadequate to analyse some compounds in environmental samples. Also, a low recovery rate meant that the method is validated but will require a lot of starting material – higher sample volume as well as higher solvent volume – and an instrument with higher sensitivity.

Finally, it is important to note that environmental water samples are complex matrixes which means that co-elution of different organic compounds can occur and modify the ionisation performance by enhancing or suppressing the concentrations of the targeted substances. For this reason, to validate a reliable HPLC-ESI-MS-MS method, matrix effects should be studied (Taylor 2005). Numerous studies evaluated the matrix effect on the detection of anticancer drugs. For example, in one study, signal enhancement was apparent for cyclophosphamide and methotrexate, while signal suppression was detected for gemcitabine, epirubicin, ifosfamide and irinotecan (Rabii et al. 2014). These matrix effects should not be neglected, and effort should be made to reduce them such as using isotopically labelled internal standards, ameliorating the sample preparation or reducing the flow rate (Kloepfer et al. 2005). Whenever possible, the deuterated form of the compounds analysed has been employed (e.g. cyclophosphamide-d6, paclitaxel-d5, etc.) or isotopically labelled compounds such as ^13^C-phenacetin and ^13^C_3_-caffeine. Also, in one study, matrix-matched calibration was performed to correct the matrix effect (Kot-Wasik et al. 2016). For sample preparation, centrifuging and filtration always preceded SPE in order to remove suspended solids and bacteria. Filters used consisted of glass microfiber, cellulose nitrate, cellulose acetate or nylon filters with pore size reaching 0.45 μm. Further clean-up of the samples was achieved when using two cartridges in tandem (Liu et al. 2010; Yin et al. 2010a; Camacho-Muñoz and Kasprzyk-Hordern 2015).

## Research gaps and recommendations

This systematic review was developed in accordance with the PRISMA 2009 checklist (Supplementary Information 2). The main purpose of this research was to review publications in which anticancer drugs were analysed in the aquatic environment. A small number of countries were represented in examining anticancer compounds and studies focused on one specific area in each country.

With the growing interest of detecting anticancer drugs in the aquatic environment, several extraction and analytical methods were established with most combinations applying SPE followed by LC-MS-MS. Overall, the majority of anticancer drugs analysed and detected in the 75 included studies are cyclophosphamide, tamoxifen, ifosfamide and methotrexate which correlates with them being among the most frequently used drugs in cancer therapy.

In summary, the main recommendations extrapolated from this systematic review are as follows:

- Research needs to be broadened, geographically speaking, in order to understand the scope of the problem.
- Guidelines for sampling strategies are required to submit reliable and representative results.
- The choice of anticancer drugs to be analysed in the water should depend on the consumption rate of this specific drug and whether it is administered in the addressed hospital or in the surveyed area.
- Researchers should roughly predict the different compounds (parent compound or metabolite) expected to be present in their samples based on the percentage of excretion of the anticancer drug, its elimination form and its stability in the water.
- More work needs to be done in terms of developing specific and efficient analytical methods, in order to reach higher sensitivity and selectivity of some anticancer drugs.
- The different variability factors should be taken into consideration while discussing the results obtained in order to be certain whether the compound exist in the aquatic environment or not.

## Electronic supplementary material

ESM 1 (PDF 1434 kb)

ESM 2 (PDF 113 kb)
