# Supplementary material for: Occurrence of anticancer drugs in the aquatic environment: a systematic review
Source: Environ Sci Pollut Res Int. 2019 Dec 12;27(2):1339–47. doi: 10.1007/s11356-019-07045-2 (PMC6994516; doi:10.1007/s11356-019-07045-2)
Supplement: Supplementary file 1 — (PDF 1434 kb) [file 11356_2019_7045_MOESM1_ESM.pdf]

Occurrence of anticancer drugs in the aquatic environment: a systematic review

Carla NASSOUR[1], Stephen J. BARTON[1], Shereen NABHANI-GEBARA[1], Yolande SAAB[2], James BARKER[1]*

[1] Kingston University, Penrhyn Road, Kingston Upon Thames, United Kingdom, KT1 2EE

[2] Lebanese American University, Byblos, Lebanon

Author Information

Corresponding Author

*Phone: +44 785 242 9113; email: k1801084@kingston.ac.uk

Table 1: Characteristics of the included studies

Ref	Year	Country	Compound	Sample source (n=number of samples)	Extraction	Separation and Detection	Concentration Range (ng/L)	LOD (ng/L)	LOQ (ng/L)	%R
1	1996	Germany	Ifosfamide	Hospital's main sewage pipe (n=1)	SPE – SiOH SPE cartridges	GC-MS	24	6-7	-	30-39
			Cyclophosphamide				146			
2	1996	Germany	Cyclophosphamide	Hospital's main sewage water (n=7)	SPE – SiOH SPE cartridges	GC-MS	19-4500	6	-	30
				Influent of a municipal WWTP (n=22)			<6-143<br>n.d. (n=1)			
				Effluent of a municipal WWTP (n=24)			6-17<br>n.d. (n=3)			
3	1997	Germany	Ifosfamide	Hospital of Tumour Biology effluents (n=7)	SPE – RP-18 cartridges	GC-MS	<6-1914	7	-	-
				WWTP Influent (n=12)			<6-29			
				WWTP Effluent (n=12)			<6-43			
				Countryside effluents (n=NA)			<6			
4	2001	Germany	Ifosfamide	Groundwater (n=105)	SPE – PPL Bond-Elut material	LC-ESI-MS-MS	n.d.	4.2-10	-	71-73
			Cyclophosphamide				n.d.			
5	2003	Italy	Cyclophosphamide	River (n=8)	SPE – Lichrolut EN	LC-MS-MS	n.d.	0.0074	0.022	99
6	2003	United Kingdom	Cyclophosphamide	WWTP influent and effluent (n=30)	SPE – C18	LC-MS	n.d.	23	-	89
7	2003	United Kingdom	Tamoxifen	Effluent of WWTP (n=3)	SPE – StrataX	LC-ESI-MS-MS	<10	10	-	42
				Upstream of WWTP (n=1)			<10			
				Downstream of WWTP (n=1)			<10			
8	2004	Austria	5-Fluorouracil	Hospital's wastewater (n=9)	SPE – Env+	CE - Diode array detector	20000-122000	1700	8600	87.8

9	2004	United Kingdom	Tamoxifen	Mersey estuary (n=6)	SPE – StrataX	LC-ESI-MS-MS	13 (n=1)<br><4 (n=5)	4	-	42
				Tyne estuary (n=6)			<4 (n=6)			
				Thames estuary (n=2)			<4 (n=2)			
				Belfast Lough estuary (n=3)			35-71 (n=2)<br><4 (n=1)			
				Tees estuary (n=5)			23 (n=1)<br><4 (n=4)			
10	2004	United Kingdom	Tamoxifen	Upstream of WWTP (n=15)	SPE – StrataX	LC-ESI-MS-MS	<10	10	-	42
				Final effluent of WWTP (n=45)			<50-42			
				Downstream of WWTP (n=15)			<10			
11	2005	United Kingdom	Tamoxifen	Raw wastewater (n=3)	SPE – StrataX	LC-ESI-MS-MS	143-215	10	-	42
				Pre-UV WW (n=3)			376-740			
				Treated WW (n=3)			146-369			
				Surface water (n=18)			27-212			
12	2005	Italy	Cyclophosphamide	Effluents of WWTPs (n=8)	SPE – LiChrolut EN and Oasis MCX	RP-LC-MS-MS	2.1 (n=1)<br>9.0 (n=1)<br>n.d. (n=6)	-	0.83-1.9	76-106
			Methotrexate	Effluents of WWTPs (n=8)			12.6 (n=1)<br>n.d. (n=7)			
13	2006	Romania	Cyclophosphamide	Somes River from 4 sites (n=4)	SPE – Oasis	GC-MS	64.8 ± 8.0	-	30	68.3
							46.1 ± 7.2			
							<30			
							<30			
14	2006	Austria	Epirubicin	Hospital effluents (n=28)	SPE – C8	RP-LC Fluorescence detection	100-1400	50-60	-	85.2-87.6
			Doxorubicin				100-500			
			Daunorubicin				n.d.			
15	2006	Austria	5-Fluorouracil	Raw WW of hospital (n=98)<br>Treated WW of hospital (n=NA)	SPE – StrataX and C8	CE-Diode array detection (5-FU)	<8600-124000 (untreated)<br><LOD (treated)	-		260-8600
			Epirubicin				n.d.			

			Doxorubicin			LC-Fluorescence detection (Anthracycline)	<260-1350 (untreated) <LOD (treated)			
			Daunorubicin				n.d.			
16	2006	Switzerland	Cyclophosphamide Ifosfamide	WWTPs influent (n=5)	SPE – reusable cartridges (macroporous polystyrene divinylbenzene adsorbent)	GC-MS	(CP) 2-11 (IF) 1.4-<15	0.02-2	-	74-102
				WWTPs effluent (n=4)			(CP) 2-10 (IF) 1.7-6			
				Surface Water (n=5)		LC-MS-MS	(CP) 0.05-0.17 (IF) <0.05-0.14			
17	2006	Switzerland	Tamoxifen	Wastewaters from 1 hospital, 1 residential area, and 2 WWTPs (n=37)	LLE (TAM) SPE (5-FU) – Env+	GC-MS	(TAM) 1-4	1-30	4-90	73-81
							(TAM) n.d. in treated WW			
			5-Fluorouracil				(5FU) n.d.			
18	2007	United Kingdom	Tamoxifen	Sewage outfall (n=NA)	SPE – Oasis HLB	LC–ESI-MS–MS	<0.003	0.003	0.009	49.5-62.3
				Upstream of sewage (n=NA)			<0.003			
				Downstream of sewage (n=NA)			<0.003			
19	2007	United Kingdom	Tamoxifen	Sea (n=NA)	SPE – Strata X	LC-MS-MS	<0.03	0.03	0.08	15-29
				Tap water (n=NA)			<0.03			
				River (n=NA)			<0.03			
				Wastewater effluent (n=NA)			<0.03			
20	2008	Romania	Cyclophosphamide	Before upgrade of WWTP (n=3)	SPE – Oasis HLB	GC-MS	45-65 (n=2) <10 (n=1)	-	10	55-110
				After upgrade of WWTP (n=3)		GC-ITMS	n.d. (n=3)			
21	2008	United Kingdom	Tamoxifen	3 WWTPs influents (n=5)	SPE – Oasis HLB	LC-MS-MS	0.1-1.3	0.003	0.009	52
				3 WWTPs effluents (n=5)			0.1-0.5			
				Upstream WWTP (n=5)			n.d.			
				Downstream WWTP (n=5)			n.d.			
22	2008	Sweden	Cyclophosphamide	Influent of WWTP (n=4)	SPE – MCX and MAX	LC-MS-MS	<25	3.8-9.1	-	87-92

				Effluent of WWTP (n=4)			<15			
				Ozone-treated effluent (n=4)			<15			
				MBR treated effluent (n=4)			<20			
23	2008	Switzerland	5-Fluorouracil	Hospital WW (n=17)	SPE – Isolute Env+	HILIC-MS-MS	>5-27 (n=6)	0.00003-0.00048	5-9	-
			Gemcitabine				>0.9-38 (n=6)			
			2',2'-difluorodeoxyuridin (dFdU)				>9-840 (n=12)			
24	2009	Canada	Cyclophosphamide<br>Methotrexate	Influent of WWTP (n=NA)	On-line SPE – Strata X	LC-ESI(±)-MS-MS	(CP) <5-9<br>(MTX) <11-59	5-16	14-47	55-148
				Effluent of WWTP (n=NA)			(CP) <9<br>(MTX) <16			
				River (n=NA)			(CP) <9<br>(MTX) <16			
25	2009	Spain	Tamoxifen	SW (n=15)	SPE – Oasis HLB	LC-ESI-MS-MS	<0.3	0.3-1	1-4	66-149
				WW influent (n=3)			<1			
				WW effluent (n=3)			<0.4			
26	2009	Switzerland	5-Fluorouracil	Hospital WW (n=25)	SPE – ENV+	LC-MS-MS	<5-27	-	0.9-9	54-118
			Gemcitabine				<0.9-38			
			dFdU				<9-839			
27	2009	Australia	Cyclophosphamide<br>Ifosfamide	Post-reverse osmosis water (n=6)	SPE – Strata X	LC-MS-MS	(CP) <5<br>(IF) <25	-	5-100	96-167
				Secondary water (n=6)			(CP) <100<br>(IF) <100			
28	2009	France	5-Fluorouracil	Hospital effluent (n=14)	SPE – Env+	GC-MS-MS	90-4000 (n=12)<br>n.d. (n=2)	-	-	100
29	2009	Australia	Cyclophosphamide<br>Ifosfamide	Hospital WW (n=4)	SPE – Oasis HLB	LC-MS-MS	<LOQ	-	100-2000	100
				WWTP influent (n=4)						
30	2009	Sweden	Cyclophosphamide	Influent WW (n=4)	Bag-SPE – XAD-2 resin<br>SPE – Oasis HLB	UPLC-MS-MS	Bag-SPE: 150<br>SPE: 114	20	60	41.1-73.2
				Effluent WW (n=4)			Bag-SPE: 114<br>SPE: 33.3			
31	2009	China	Vincristine		SPE – Oasis HLB		<20	2-20	-	51-105

			Azathioprine	Hospital effluents (n=65)		UPLC-ESI-MS-MS	9-32 (n=3)<br><5 (n=62)			
			Ifosfamide				4-10647 (n=38)<br><2 (n=27)			
			Cyclophosphamide				6-2000 (n=47)<br><2 (n=18)			
			Methotrexate				4-4689 (n=14)<br><2 (n=51)			
			Etoposide				6-380 (n=15)<br><5 (n=50)			
			Procarbazine				<5			
			Doxorubicin				<10			
			Doxorubicinol				<10			
32	2009	Canada	Cyclophosphamide<br>Methotrexate	Drinking water (n=3)	On-line SPE – Strata X	LC-MS-MS	(CP) <1<br>(MTX) <1	1-2	-	61-70
				Surface water (n=3)			(CP) <1<br>(MTX) <2			
33	2010	China	Raloxifene (1)<br>Letrozole (2)<br>Anastrozole (3)<br>Exemestane (4)<br>Tamoxifen (5)<br>Toremifene (6)<br>Clomiphene (7)<br>N-desmethyltamoxifen (8)<br>Mifepristone (9)<br>Finastride (10)	WWTP Influent (n=3)	SPE – Oasis HLB	LC-MS-MS	(1) 0.8-1.52 (n=3)<br>(2) 0.28-0.8 (n=3)<br>(3) 0.12-0.32 (n=3)<br>(4) <MDL<br>(5) 0.28 (n=1)<br>(6) 0.58 (n=1)<br>(7) 0.18 (n=1)<br>(8) <MDL<br>(9) 0.4-1.62 (n=3)<br>(10) 0.28-1 (n=2)	-	0.05-2	-
				WWTP Effluent (n=2)			(1) 0.26-0.4 (n=2)<br>(2) 0.27-0.6 (n=2)<br>(3) 0.3 (n=1)<br>(4) <MDL<br>(5) <MDL<br>(6) <MDL<br>(7) <MDL<br>(8) <MDL<br>(9) 0.7-0.75 (n=2)<br>(10) 0.1 (n=1)			

				Hospital effluent (n=63)			(1) 2.2-6.74 (n=2)<br>(2) 0.2-2.38 (n=52)<br>(3) 0.3-3.7 (n=52)<br>(4) <MDL<br>(5) 0.2-8.2 (n=23)<br>(6) <MDL<br>(7) <MDL<br>(8) <MDL<br>(9) 0.64-195 (n=17)<br>(10) 0.32-4.36 (n=34)			
34	2010	Spain	Tamoxifen	Drinking Water (n=6)	On-line SPE – Hysphere Resin GP	LC-ESI-MS-MS	n.d.	0.78-5.41	2.1-18.05	74.16-88.61
				Surface Water (n=12)			n.d.			
				WW effluent (n=6)			n.d.			
35	2010	China	Methotrexate	WWTP influents and effluents (n=10)	SPE – Oasis HLB	LC-ESI-MS-MS	1.6-18.1 (influent)<br>n.d. (effluent)	0.6-7	1.7-20	51-104
			Azathioprine				n.d.			
			Doxorubicinol				n.d.			
			Doxorubicin				n.d.			
			Cyclophosphamide				8.5-14.5			
			Ifosfamide				9.0-16.4			
			Vincristine				n.d.			
			Etoposide				n.d.			
			Procarbazine				n.d.			
36	2010	Spain	Cyclophosphamide	4 WW effluents (n=5 each)<br>4 River water (n=5 each)	SPE – Oasis HLB	LC-MS-MS	n.d	<5	-	81-84
			Ifosfamide				n.d			
			Tamoxifen				n.d.			
37	2011	Canada	Cyclophosphamide<br>Methotrexate	Raw sewage of WWTP (n=NA)	On-line SPE – Strata X	LC-APPI-MS-MS	(CP) <11<br>(MTX) not ionised	5-11	-	-
						LC-APCI-MS-MS	(CP) <7<br>(MTX) not ionised			
						LC-ESI-MS-MS	(CP) 12<br>(MTX) <11			
38	2011	Finland	Cyclophosphamide	WW effluent (n=1)	SPE – Oasis HLB and Strata X	UPLC-MS-MS	<26	11	26	99-110

39	2011	United Kingdom	Cyclophosphamide<br>Ifosfamide	WWTP 1 (Pre UV) (n=1)	SPE – Strata X	LC-ESI-MS-MS	(CP) 3.7<br>(IF) <0.05	0.03-0.12	0.11-0.24	-
				WWTP 1 (Post UV) (n=1)			(CP) 3.5<br>(IF) <0.05			
				WWTP 2 effluent (n=1)			(CP) 0.19<br>(IF) 0.05			
40	2011	Spain	Tamoxifen	River (n=13)	SPE – Oasis HLB	UHPLC-MS-MS	<0.05	0.02	0.05	9.3
				Tributaries (n=13)			<0.05-19.57			
41	2011	Spain	Cytarabine	(1) River water (n=3)<br>(2) Effluent WW (n=3)<br>(3) Influent WW (n=3)	SPE – Oasis HLB	HPLC-MS-MS	(1) 13<br>(2) 14<br>(3) 9.2	0.1-38	0.3-128	11-99
			Cyclophosphamide				(1) <1.7<br>(2) <2.3<br>(3) <2.1			
			Docetaxel				(1) <1.5<br>(2) <1.7<br>(3) <1.9			
			Doxorubicin				(1) <5.3<br>(2) <4.3<br>(3) 4.5			
			Epirubicin				(1) <3.5<br>(2) <0.7<br>(3) <3.8			
			Etoposide				(1) <2.2<br>(2) 3.4<br>(3) 15			
			5-Fluorouracil				(1) <34<br>(2) <21<br>(3) <38			
			Gemcitabine				(1) 2.4<br>(2) 7.0<br>(3) 9.3			
			Ifosfamide				(1) <1.3<br>(2) 1.2<br>(3) 3.5			
			Irinotecan				(1) <0.9<br>(2) <1.0<br>(3) <1.1			

			Methotrexate				(1) <0.1<br>(2) <0.1<br>(3) <0.1			
			Mitomycin C				(1) <2.2<br>(2) <2.0<br>(3) <1.7			
			Paclitaxel				(1) <0.3<br>(2) <0.2<br>(3) <0.3			
			Vinorelbine				(1) <4.0<br>(2) 9.1<br>(3) <5.2			
42	2012	Spain	Cyclophosphamide<br>Epirubicin	Urban effluent (n=1)	SPE – Oasis HLB	LC-MS-MS	(CP) <0.35<br>(Epi) <2.77-24800	0.35-2.77	-	43-107
				Hospital effluent (n=1)			(CP) 0.35-5730<br>(Epi) <2.77			
				WWTP influent (n=3)			(CP) <0.35-13100<br>(Epi) <2.77			
				WWTP effluent (n=3)			(CP) <0.35<br>(Epi) <2.77			
43	2012	Spain	Tamoxifen	River upstream (n=6)	SPE – HySphere Resin GP	LC-MS-MS	<1.5	1.5-5.41	5.05-18	-
				River downstream (n=6)			<1.5			
				WWTP effluent (n=6)			<5.41			
44	2012	Spain	Tamoxifen	River downstream WWTP (n=8)	SPE – Oasis HLB	LC-MS-MS	0.3-1.3	-	-	-
				River downstream WWTP (n=3)			0.2-1.3			
				River upstream WWTP (n=8)			0.3-1.8			
45	2012	Italy	Tamoxifen	Hospital effluent (n=4)	SPE – Oasis HLB	LC-MS-MS	<1	1-2	-	65-145
				Hospital effluent (n=4)			<1			
				Hospital effluent (n=4)			<2			
				WWTP influent (n=4)			<1			
				WWTP effluent (n=4)			<1			
46	2012	Spain	Tamoxifen	River (n=NA)	Online-TFC	LC-ESI-MS-MS	22.4-22.8	0.38	1.27	86.5
47	2012	Spain	Tamoxifen	River (n=9)	Online-TFC	LC-ESI-MS-MS	12.4-20.1	-	<10	>80
				Tributaries (n=15)			22.4-26.8			

48	2012	Sweden	Flutamide	WWTPs effluents (n=6)	SPE – Oasis HLB	LC-MS-MS	<5.1	-	5.1-55	68-100
			Tamoxifen				13-24<br><5 (n=1)			
			Megestrol				<55			
49	2012	Spain	Tamoxifen	Surface water (n=4)	SPE – Oasis HLB	LC-MS-MS	0.01-1.34<br>n.d. (n=1)	-	-	-
				Surface water (n=4)			0.16-115.04<br>n.d. (n=1)			
50	2012	Spain	Tamoxifen	GW (n=13)	On-line SPE – HySphere Resin GP	LC-ESI-MS-MS	9.25-88.3	0.63-1.51	2.10-5.05	74.16-88.61
				GW (n=5)			11.2-39.3			
				GW (n=13)			18.9-223			
				River (n=1)			17.6			
51	2013	Spain	Gemcitabine	WWTP influent (n=8)	On-line SPE – PLRP-s	LC-MS-MS	<9.3	0.1-54	0.4-180	60-119
			Temozolomide				<50			
			Methotrexate				2.1-20.1			
			Irinotecan				<4.5			
			Imatinib				<180			
			Ifosfamide				7.3-43.3 (n=4)<br><2 (n=4)			
			Cyclophosphamide				<3			
			Erlotinib				<3.4			
			Etoposide				<65			
			Doxorubicin				<2.5			
			Capecitabine				8.2-27 (n=7)<br><5 (n=1)			
			Tamoxifen				3.5-17.2 (n=4)<br><3.4 (n=1)<br><1 (n=3)			
			Paclitaxel				<4.4			
			Hydroxymethotrexate				<5.2			
			Desmethylhydroxytamoxifen				<5			
			Hydroxytamoxifen				<5			
			Hydroxypaclitaxel				4.4 (n=1)<br><1.1 (n=7)			
52	2013	France<br>Portugal	Ciprofloxacin<br>Azathioprine	Hospital effluent 1 (n=3)	SPE – Oasis HLB	UPLC-MS-MS	CIP 679.1±21.1<br>AZA 21.6±2.1	0.8-24	2.4-80	46-129

		Spain	Cyclophosphamide<br>Ifosfamide<br>Tamoxifen<br>Docetaxel<br>Paclitaxel<br>Etoposide<br>Vincristine<br>Methotrexate				CY 43.4±4.5<br>IF 31.5±7.5<br>TAM 59.5±3.5<br>DOC n.d.<br>PAC n.d.<br>ETO 97.5±19.1<br>VIN 49.1±7.2<br>MTX < 5.9			
				Hospital effluent 2 (n=3)			CIP 2,684.1±82.1<br>AZA 14.5±2<br>CY 35.9±5.5<br>IF n.d.<br>TAM 26.3±1.1<br>DOC n.d.<br>PAC n.d.<br>ETO n.d.<br>VIN <24.5<br>MTX n.d.			
				Hospital effluent 3 (n=3)			CIP 14725±2259<br>AZA 24.5±2.5<br>CY <3.6<br>IF n.d.<br>TAM 94.3±2.3<br>DOC 97.7±21<br>PAC 99.7±13.1<br>ETO 406±68.1<br>VIN <24.5<br>MTX n.d.			
				Hospital effluent 4 (n=3)			CIP 9278.5±167<br>AZA 187.9±1.5<br>CY 200.7 ±0.9<br>IF 227.9±1.3<br>TAM 133.4±2.6<br>DOC n.d.<br>PAC n.d.<br>ETO n.d.<br>VIN n.d.<br>MTX n.d.			

				WW influent 1 (n=3)			CIP 1557±163.1<br>AZA 18.2±2.4<br>CY 25.5±2<br>IF n.d.<br>TAM 58.3±4.7<br>DOC 175.1±37<br>PAC n.d.<br>ETO 83±17<br>VIN 22.9±0.1<br>MTX 23±0.4			
				WW influent 2 (n=3)			CIP 1172.1±74<br>AZA 19.1±1.2<br>CY n.d.<br>IF n.d.<br>TAM 51±0.7<br>DOC n.d.<br>PAC n.d.<br>ETO n.d<br>VIN n.d.<br>MTX <5.9			
				WW influent 3 (n=3)			CIP 3200±2.4<br>AZA n.d.<br>CY n.d.<br>IF 130.1±1.3<br>TAM 30±2.8<br>DOC n.d.<br>PAC n.d.<br>ETO n.d.<br>VIN n.d.<br>MTX n.d.			
53	2013	Slovenia	5-Fluorouracil	Hospital A effluent (n=2)	SPE – Isolute Env+	GC-MS-MS	35-92	0.16-0.48	0.54-1.6	-
				Hospital B effluent (n=2)			<0.48			
				WWTPs influent (n=2)			4.7-14			
				WWTPs effluent (n=4)			<0.16			
				Surface water (n=3)			<0.16			

54	2013	United Kingdom	Tamoxifen	WWTP influent (n=2)	SPE – Oasis HLB	LC-MS-MS	16-46	0.003	0.009	49.5-62.3
				WWTP effluent (n=2)			8-32			
55	2013	France	Cyclophosphamide<br>Ifosfamide	Surface water (n=7)	On-line SPE – Strata X	LC-MS-MS	n.d.<br>n.d.	4	11	88.7-110
				Tap water (n=2)			n.d.<br>n.d.			
56	2014	Canada	Gemcitabine<br>Methotrexate<br>Ifosfamide<br>Cyclophosphamide<br>Irinotecan<br>Epirubicin	WWTPs influent (n=4)	On-line SPE – Hypersil Gold PFP	LC-MS-MS	(GCA) <20<br>(MTX) 17-60<br>(IF) <4<br>(CP) 17-22 (n=2)<br>(CPT-11) <6<br>(EPI) <18	4-20	13-60	47-90
				WWTPs effluent (n=5)			(GCA) <20<br>(MTX) 13-53 (n=4)<br>(IF) <4<br>(CP) 18-21 (n=2)<br>(CPT-11) <6<br>(EPI) <18			
57	2014	Spain	(1)Cyclophosphamide<br>(2)Cytarabine<br>(3)Docetaxel<br>(4)Doxorubicin<br>(5)Epirubicin<br>(6)Etoposide<br>(7)5-Fluorouracil<br>(8)Gemcitabine<br>(9)Ifosfamide<br>(10)Irinotecan<br>(11)Methotrexate<br>(12)Mitomycin C<br>(13)Paclitaxel<br>(14)Vinorelbine	Influent WW (n=48)	SPE – Oasis HLB	LC-MS-MS	(1) <2.13<br>(2) 44.4-464<br>(3) <1.89<br>(4) <4.15<br>(5) <3.75<br>(6) 15.1-46.8<br>(7) <38.4<br>(8) 39.3-52.1<br>(9) 6.49-19.1<br>(10) <1.12<br>(11) 7.30-55.8<br>(12) <1.66<br>(13) <0.26<br>(14) <5.18	0.08-38.4	0.27-128	11-105

				Effluent WW (n=48)			(1) <2.29<br>(2) 9.9-190<br>(3) <1.65<br>(4) 20.3-42.4<br>(5) <0.7<br>(6) <2.95<br>(7) <21.1<br>(8) 64.6-88.4<br>(9) 4.14-15.6<br>(10) <1.04<br>(11) <0.08<br>(12) <1.97<br>(13) 1.4-2.19<br>(14) 44.1-170			
58	2014	Spain	Tamoxifen	River (n=549)	SPE – Strata X	LC-ESI-MS-MS	3.5-11.7 (n=6)	2	3	75
59	2014	Spain	(1)Cyclophosphamide<br>(2)Ifosfamide<br>(3)Capecitabine<br>(4)Epirubicin<br>(5)Irinotecan<br>(6)Goserelin<br>(7)Megestrol<br>(8)Prednisone	Hospital A effluent (n=20)	SPE – Oasis HLB	LC-MS-MS	(1) <4-4720<br>(2) <6-86200<br>(3) 15-490<br>(4) <45<br>(5) <4-730<br>(6) <16-350<br>(7) <3-1260<br>(8) <12-210	-	3-45	44-104
				WWTP influent (n=5)			(1) <4-10<br>(2) <6<br>(3) <15<br>(4) <45<br>(5) <4<br>(6) <16<br>(7) <3-150<br>(8) <12			
				WWTP effluent (n=5)			(1) <4-5<br>(2) n.d.<br>(3) n.d.<br>(4) n.d.<br>(5) n.d.<br>(6) n.d.<br>(7) <3-20			

							(8) n.d.			
				Hospital B effluent (n=10)			(1) <4-20<br>(2) <6-2690<br>(3) <15<br>(4) <45-60<br>(5) <4<br>(6) <16<br>(7) <3-70<br>(8) <12			
				WWTP influent (n=10)			(1) <4<br>(2) <6<br>(3) <15<br>(4) <45<br>(5) <4<br>(6) <16<br>(7) <3-220<br>(8) <12			
				WWTP effluent (n=10)			(1) n.d.<br>(2) n.d.<br>(3) n.d.<br>(4) n.d.<br>(5) n.d.<br>(6) n.d.<br>(7) <3<br>(8) n.d.			
60	2014	Spain	(1)Azathioprine<br>(2)Ciprofloxacin<br>(3)Cyclophosphamide<br>(4)Docetaxel<br>(5)Etoposide<br>(6)Ifosfamide<br>(7)Methotrexate<br>(8)Paclitaxel<br>(9)Tamoxifen<br>(10)Vincristine	Hospital WW (n=3)	SPE – Oasis HLB	UPLC-MS-MS	(1) <12.7-90<br>(2) 3089-14826<br>(3) <3.6-43<br>(4) 61-79 (n=2)<br>(5) <80-714<br>(6) n.d.<br>(7) 6-19 (n=2)<br>(8) <18.4-100<br>(9) 36-170<br>(10) <24.5 (n=1)	0.2-28.8	0.8-96.2	56.5-129.7
				WW influent (n=3)			(1) 19-20 (n=2)<br>(2) 1172-1558<br>(3) 8-26			

							(4) 65-219<br>(5) <96.2 (n=2)<br>(6) n.d.<br>(7) <4.8 -23<br>(8) 18 (n=1)<br>(9) 15-58<br>(10) n.d.			
				WW effluent (n=3)			(1) n.d.<br>(2) 36-147<br>(3) 7-25<br>(4) n.d.<br>(5) n.d.<br>(6) n.d.<br>(7) 6 (n=1)<br>(8) <8.7 (n=1)<br>(9) 11-42<br>(10) n.d.			
				SW before WWTP (n=3)			(1) n.d.<br>(2) 8-56<br>(3) n.d.<br>(4) n.d.<br>(5) n.d.<br>(6) n.d.<br>(7) n.d.<br>(8) n.d.<br>(9) 12-36<br>(10) n.d.			
				SW after WWTP (n=3)			(1) n.d.<br>(2) 7-103<br>(3) <0.9-20<br>(4) n.d.<br>(5) n.d.<br>(6) n.d.<br>(7) 5 (n=1)<br>(8) n.d.<br>(9) 25-38<br>(10) n.d.			

61	2014	Spain	Methotrexate<br>Ifosfamide<br>Cyclophosphamide<br>Irinotecan<br>Doxorubicin<br>Capecitabine<br>Tamoxifen<br>Endoxifen<br>Hydroxytamoxifen<br>Hydroxypaclitaxel	WW influent (n=24)	On-line SPE – PLRP-s cartridges	LC-MS-MS	MET 2.6-18.1 (n=6)<br>IF 2.2-27.9 (n=9)<br>CP 2.4-43.8 (n=12)<br>IRI 8.8-21.3 (n=4)<br>DOX 2.5-2.7 (n=2)<br>CAP 5.6-72.6 (n=11)<br>TAM 177.6-180.6 (n=4)<br>OH-D-TAM <LOD<br>OH-TAM <LOD<br>OH-PAC 3.7-18.5 (n=4)	-	1.1-4.5	71-119
				WW effluent (n=31)			MET 2-19.4 (n=4)<br>IF 2.5-19.4 (n=12)<br>CP 2.5-100 (n=20)<br>IRI 16.8 (n=1)<br>DOX <LOD<br>TAM 102-147 (n=12)<br>OH-D-TAM 91.6 (n=1)<br>OH-TAM 5.8-164 (n=3)<br>OH-PAC 3.7 (n=1)			
62	2015	Slovenia	Cyclophosphamide<br>Ifosfamide	Hospital WW (n=10)	SPE – Oasis HLB	GC-MS	CP 15-22000 (n=7)<br>IF 48-6800 (n=3)	0.36-2.8	1.2-9.4	93-99
				WWTP influent (n=4)			CP 19-27 (n=2)<br>IF n.d.			
				WWTP effluent (n=4)			CP 17 (n=1)<br>IF n.d.			
63	2015	Spain	Cisplatin	Hospital WW (n=1)	-	ZIC-HILIC-ICP-MS	14.4	0.1726	0.5753	95-101
				WWTP influent (n=1)			7.9			
				WWTP effluent (n=1)			5.9			
		Slovenia	Cisplatin	Hospital WW (n=1)	-	ZIC-HILIC-ICP-MS	352			
				WWTP influent (n=1)			23.3			
				WWTP effluent (n=1)			12.8			

64	2015	Japan	(1)Bicultamide<br>(2)Capecitabine<br>(3)Cyclophosphamide<br>(4)Doxifluridine<br>(5)Tamoxifen<br>(6)Tegafur	River main stream (n=24)	SPE – Oasis Max	UPLC-MS-MS	(1) 32<br>(2) 2<br>(3) 2<br>(4) n.d.<br>(5) n.d.<br>(6) n.d.	0.1-0.2	0.2-0.8	63-124
				Tributary (n=16)			(1) 30<br>(2) 1<br>(3) 3<br>(4) n.d.<br>(5) n.d.<br>(6) n.d.			
				WWTP effluent (n=10)			(1) 245<br>(2) 6<br>(3) 10<br>(4) n.d.<br>(5) n.d.<br>(6) 23			
				WWTP effluent ozonation (n=10)			(1) 5<br>(2) 2<br>(3) 3<br>(4) n.d.<br>(5) n.d.<br>(6) n.d.			
65	2015	United Kingdom	(1)Ifosfamide<br>(2)3-N-Dechloroethyl ifosfamide	WW effluent (n=NA)	SPE – Oasis HLB - MAX	cLC-MS-MS	(1) <0.31 E1<br>(1) <0.29 E2<br>(2) <11.1 E1<br>(2) <9.79 E2	0.04-3.33	0.14-11.10	1.5-119.2
				Surface water (n=NA)			(1) <0.14 E1<br>(1) <0.15 E2<br>(2) <3.62 E1<br>(2) <3.78 E2			
66	2016	United Kingdom	Azathioprine<br>Methotrexate<br>Ifosfamide<br>Tamoxifen	WW influent (n=3)	SPE – Oasis HLB	UPLC-MS-MS	<1.36<br><23.45<br><1.53<br><3.5	0.08-14.52	0.4-72.6	66.4-114.1
				WW effluent (n=3)			<1.2<br><29.83			

							<1.22<br><3.82			
				Surface water (n=3)			<0.55<br><20.24<br><0.4<br><72.6			
67	2016	Slovenia	(1)Cyclophosphamide<br>(2)Ifosfamide<br>(3)Carboxy-CP<br>(4)Keto-CP<br>(5)N-decl-CP	Hospital WW (n=7)	SPE – Oasis HLB and Env+	GC-MS	(1) 76-2686<br>(2) 26-47<br>(3) 213-13202<br>(4) <13.1-178<br>(5) 60-2099	2-23	6.7-77.7	58.1-102.9
				WWTP influent (n=1)			n.d.			
				WWTP effluent (n=1)			n.d.			
68	2016	Northern and Central Europe	(1)Ifosfamide<br>(2)3-N-Dechloroethyl ifosfamide	WWTP influent (n=6)	SPE – Oasis HLB - Max	cUHPSFC-MS-MS	(1) <0.51 E1<br>(1) <0.58 E2<br>(2) <0.46 E1<br>(2) <0.22 E2	0.51-8.62	1.54-28.7	>70
				WWTP effluent (n=6)			(1) <0.51 E1<br>(1) <0.54 E2<br>(2) <1.35 E1<br>(2) <8.62 E2			
69	2016	Slovenia	(1)Platinum<br>(2)Cyclophosphamide (CP)<br>(3)Ifosfamide<br>(4)Keto-cyclophosphamide<br>(5)N-dechloroethyl-CP<br>(6)Carboxy-CP<br>(7)5-Fluorouracil<br>(8)Gemcitabine<br>(9)Methotrexate<br>(10)Hydroxymethotrexate<br>(11)Irinotecan<br>(12)Erlotinib<br>(13)Capecitabine<br>(14)Endoxifen<br>(15)(Z)-4-hydroxytamoxifen	Hospital WW (n=2)	On-line SPE<br>SPE – Oasis HLB, Isolute Env+ and PLRP-s crosslinked styrene-divinylbenzene polymer	GC-MS<br>ICP-MS<br>LC-MS-MS	(1) 226-352<br>(2) 1080-22100<br>(3) <4.8-48<br>(4) 270-1340<br>(5) 847-5520<br>(6) 17700-60600<br>(7) <1.6-6.9<br>(8) <0.7<br>(9) 19-3920<br>(10) <1.6-490<br>(11) <1.4-9.2<br>(12) 2-4<br>(13) <0.7-106<br>(14) <1.5<br>(15) <5-10<br>(16) <3.4-10	0.3-54	1.1-180	-

			(16)Tamoxifen	WWTP influent (n=2)			(1) 23-27<br>(2) 19-27<br>(3) <4.8<br>(4) <13<br>(5) <2<br>(6) <23<br>(7) <0.5-3.1<br>(8) <0.7-61<br>(9) 29-303<br>(10) <1.6-366<br>(11) <1.4-49<br>(12) 3.5-8.1<br>(13) <0.7-158<br>(14) <1.5-66<br>(15) <5-35<br>(16) 11-61			
				WWTP effluent (n=1)			(1) <2<br>(2) 17<br>(3) <4.8<br>(4) <13<br>(5) <2<br>(6) <23<br>(7) <0.5<br>(8) <0.7<br>(9) <0.5<br>(10) <1.3<br>(11) <0.4<br>(12) 3.8<br>(13) <0.4<br>(14) <1.5<br>(15) <0.3<br>(16) 7.1			
		Spain		Hospital WW (n=2)			(1) <2<br>(2) <2.3-32<br>(3) <4.8<br>(4) <13<br>(5) <2<br>(6) <23<br>(7) <0.5-2.1			

							(8) <0.7<br>(9) <0.6-29<br>(10) <1.6<br>(11) <1.4<br>(12) 2.4-5.5<br>(13) <0.7<br>(14) <1.5-11<br>(15) <0.7-<5<br>(16) <1-7.9			
				WWTP influent (n=2)			(1) <2<br>(2) <2.3-6<br>(3) <4.8<br>(4) <13<br>(5) <2<br>(6) <23<br>(7) <1.6-3.5<br>(8) <0.7<br>(9) 8.3-29<br>(10) <1.6<br>(11) <1.4<br>(12) 6.1-7.2<br>(13) <0.7<br>(14) <1.5-75<br>(15) <5-7.7<br>(16) 6.7-15			
				WWTP effluent (n=1)			(1) <2<br>(2) <2.3<br>(3) <4.8<br>(4) <13<br>(5) <2<br>(6) <23<br>(7) <1.6<br>(8) <0.7<br>(9) <0.5<br>(10) <1.3<br>(11) <0.4<br>(12) 3.3<br>(13) <0.5<br>(14) 14			

							(15) <1.1<br>(16) <0.9			
70	2016	Poland	Cyclophosphamide<br>Ifosfamide	WW influent (n=41)	SPE – Oasis HLB	LC-MS-MS	(CP) 9.3-33.3<br>(IF) 10.1-27.3	0.12-3.06	0.36-9.19	57.5-87.1
				WW effluent (n=42)			(CP) 1-24<br>(IF) 2.2-28			
				Untreated water (n=42)			(CP) 2.6-3.6<br>(IF) <0.18			
				Treated water (n=42)			(CP) <0.12<br>(IF) <0.12			
71	2017	United Kingdom	(1)Ifosfamide<br>(2)3-N-Dechloroethylifosfamide	WW influent (n=3)	SPE – Oasis HLB - MAX	cLC-MS-MS	(1) <0.24 E1<br>(1) <0.28 E2<br>(2) <5	0.12-5	0.41-16.7	88.5-113.4
				WW effluent (n=3)			(1) <0.23 E1<br>(1) <0.22 E2<br>(2) <5			
				River (n=3)			(1) <0.12 E1<br>(1) <0.13 E2<br>(2) <1.06			
72	2017	Spain	(1)Cyclophosphamide<br>(2)Mycophenolic acid<br>(3)Prednisone<br>(4)Capecitabine	WWTP influent (n=4)	SPE – Oasis HLB	UPLC-MS-MS	(1) 15<br>(2) 3099<br>(3) 261<br>(4) 81	2.5-120	-	57-134
				WWTP effluent (n=4)			(1) 17<br>(2) 195<br>(3) n.d.<br>(4) 13			
				WWTP effluent (n=4)	MCPS		(1) 19<br>(2) 136<br>(3) n.d.<br>(4) n.d.			
73	2017	Japan	Cyclophosphamide<br>Ifosfamide	WWTP influent (n=1)	SPE – Sep Pak PS2 and AC2	LC-MS-MS	n.d.<br>n.d.	8	-	73.7-100.4
				WWTP effluent (n=1)			n.d.<br>n.d.			
74	2017	Spain	Cyclophosphamide	2 Hospitals effluents (n=10)	On-line SPE – PLRP-s cartridges	LC-MS-MS	46-3000	0.06-22	0.3-75	-
			Ifosfamide				<0.2-4761			
			Temozolomide				<0.7)			

			Methotrexate				<0.1-4756			
			Hydroxymethotrexate				<0.2-846			
			Gemcitabine				<0.3			
			Capecitabine				<0.2-1749			
			Doxorubicin				<0.1			
			Irinotecan				<0.1-273			
			Etoposide				<3			
			Imatinib mesilate				<22-577			
			Erlotinib				<0.1			
			Paclitaxel				<0.6			
			6(α)-Hydroxypaclitaxel				<0.4			
			Tamoxifen citrate				<0.3			
			Endoxifen				<0.3			
			(Z)-4-Hydroxytamoxifen				<0.06			
75	2018	Spain	Methotrexate	WW influent (n=1)	SPE – Oasis HLB	UPLC-MS-MS	n.d.	0.00002	0.07	72
				WW effluent (n=1)			n.d.			
				Coastal water (n=28)			n.d.-3.5			
				Oceanic water (n=13)			n.d.			

E1-E2: enantiomer 1 and 2
LOD: limit of detection
LOQ: limit of quantification
%R : recovery percentage
n.d: not detected
n: number of samples
NA: not available
GW: groundwater
SW: surface water
WW: wastewater
WWTP: wastewater treatment plant
cLC: chiral liquid chromatography
cUHPSFC: chiral ultra high performance supercritical fluid chromatography
MCPS: macroporous ceramic passive sampler

Table 2: Sampling methods of the four most frequently studied compounds

Compound	Sampling type	Sampling location	Number of samples	Ref
CP	Composite sample (24h)	Hospital effluent	1	[1]
	Composite sample (24h)	Hospital effluent	7	[2]
	Sampling period of 1 h	Influent WWTP	22	[2]
	Sampling period of 1 h	Effluent WWTP	24	[2]
	NA	Groundwater	105	[4]
	Grab sample	River	8	[5]
	Time weighted composites	Sewage influent and effluent	30	[6]
	Composite sample (24h)	Effluent WWTP	8	[12]
	Grab sample	River	4	[13]
	Flow proportional (24h)	Influent WWTP	5	[16]
	Flow proportional (24h)	Effluent WWTP	4	[16]
	Grab sample	Surface water	5	[16]
	Grab sample	River	6	[20]
	Composite sample (24h)	Influent Wastewater	4	[22]
	Composite sample (24h)	Effluent Wastewater	12	[22]
	Grab sample	Influent WWTP	NA	[24]
	Grab sample	Effluent WWTP	NA	[24]
	Grab sample	River	NA	[24]
	Grab and composite samples	Treated wastewater	12	[27]
	Composite samples	Hospital effluent	4	[29]
	Composite samples	WWTP influent	4	[29]
	Composite samples (24h)	Influent WW	4	[30]
	Composite samples (24h)	Effluent WW	4	[30]
	Grab samples	Hospital effluent	65	[31]
	Grab samples	Drinking water	3	[32]
	Grab samples	Surface water	3	[32]
	Composite samples (24h)	Influent and effluent WWTP	10	[35]
	Composite samples (24h)	Effluent WWTP	20	[36]
	Grab samples	River	20	[36]
	NA	Raw sewage of WWTP	NA	[37]
	NA	Effluent WW	1	[38]
	NA	Effluent WWTP	3	[39]
	Composite sample (24h)	Influent WWTP	3	[41]
	Composite sample (24h)	Effluent WWTP	3	[41]
	Grab sample	River	3	[41]
	Composite sample (24h)	Influent WWTP	3	[42]
	Composite sample (24h)	Effluent WWTP	3	[42]
	Composite sample (24h)	Urban effluent	1	[42]
	Composite sample (24h)	Hospital effluent	1	[42]
	Time-proportional sampling	Raw wastewater	8	[51]
	NA	Hospital effluent	3	[52]
	NA	Influent WW	3	[52]
	NA	River	7	[55]
	NA	Tap water	2	[55]

	NA	Influent WWTP	4	56
	NA	Effluent WWTP	5	56
	Composite sample (24h)	Influent WW	48	57
	Composite sample (24h)	Effluent WW	48	57
	Composite sample (24h)	Hospital effluent	30	59
	Composite sample (24h)	Influent WWTP	15	59
	Composite sample (24h)	Effluent WWTP	15	59
	NA	Hospital effluent	3	60
	NA	Influent WWTP	3	60
	NA	Effluent WWTP	3	60
	NA	Surface water	6	60
	Composite sample (24h)	Influent WW	24	61
	Composite sample (24h)	Effluent WW	31	61
	Composite sample (24h) & Grab sample	Hospital effluent	10	62
	Composite sample (24h) & Grab sample	Influent WWTP	4	62
	Composite sample (24h) & Grab sample	Effluent WWTP	4	62
	Grab sample	River	40	64
	Grab sample	Effluent WWTP	20	64
	Grab sample	Hospital effluent	7	67
	Composite sample (24h)	Influent WWTP	1	67
	Composite sample (24h)	Effluent WWTP	1	67
	Grab sample	Hospital WW	4	69
	Composite sample (24h)	Influent WWTP	4	69
	Composite sample (24h)	Effluent WWTP	2	69
	Grab sample	Influent WWTP	41	70
	Grab sample	Effluent WWTP	42	70
	Grab sample	Untreated water	42	70
	Grab sample	Treated water	42	70
	Grab sample	Influent WWTP	4	72
	Grab sample	Effluent WWTP	7	72
	Grab sample	Influent WWTP	1	73
	Grab sample	Effluent WWTP	1	73
	Composite sample	Hospital effluents	10	74
IF	Composite sample (24h)	Hospital effluent	1	1
	NA	Hospital effluent	7	3
	Sampling period of 1-2h	Influent WWTP	12	3
	Sampling period of 1-2h	Effluent WWTP	12	3
	NA	Groundwater	105	4
	Flow proportional (24h)	Influent WWTP	5	16
	Flow proportional (24h)	Effluent WWTP	4	16
	Grab sample	Surface water	5	16
	Grab and composite samples	Treated wastewater	12	27
	Composite samples	Hospital effluent	4	29
	Composite samples	WWTP influent	4	29
	Grab samples	Hospital effluent	65	31
	Composite samples (24h)	Influent and effluent WWTP	10	35

	Composite samples (24h)	Effluent WWTP	20	36
	Grab samples	River	20	36
	NA	Effluent WWTP	3	39
	Composite sample (24h)	Influent WWTP	3	41
	Composite sample (24h)	Effluent WWTP	3	41
	Grab sample	River	3	41
	Time-proportional sampling	Raw wastewater	8	51
	NA	Hospital effluent	3	52
	NA	Influent WW	3	52
	NA	River	7	55
	NA	Tap water	2	55
	NA	Influent WWTP	4	56
	NA	Effluent WWTP	5	56
	Composite sample (24h)	Influent WW	48	57
	Composite sample (24h)	Effluent WW	48	57
	Composite sample (24h)	Hospital effluent	30	59
	Composite sample (24h)	Influent WWTP	15	59
	Composite sample (24h)	Effluent WWTP	15	59
	NA	Hospital effluent	3	60
	NA	Influent WWTP	3	60
	NA	Effluent WWTP	3	60
	NA	Surface water	6	60
	Composite sample (24h)	Influent WW	24	61
	Composite sample (24h)	Effluent WW	31	61
	Composite sample (24h) & Grab sample	Hospital effluent	10	62
	Composite sample (24h) & Grab sample	Influent WWTP	4	62
	Composite sample (24h) & Grab sample	Effluent WWTP	4	62
	NA	River	NA	65
	NA	Effluent WWTP	NA	65
	Grab sample	Influent WW	3	66
	Grab sample	Effluent WW	3	66
	Grab sample	River	3	66
	Grab sample	Hospital effluent	7	67
	Composite sample (24h)	Influent WWTP	1	67
	Composite sample (24h)	Effluent WWTP	1	67
	Composite sample (24h)	Influent WWTP	6	68
	Composite sample (24h)	Effluent WWTP	6	68
	Grab sample	Hospital WW	4	69
	Composite sample (24h)	Influent WWTP	4	69
	Composite sample (24h)	Effluent WWTP	2	69
	Grab sample	Influent WWTP	41	70
	Grab sample	Effluent WWTP	42	70
	Grab sample	Untreated water	42	70
	Grab sample	Treated water	42	70
	Composite sample (24h)	Influent WW	3	71
	Composite sample (24h)	Effluent WW	3	71
	Grab sample	River	3	71

	Composite sample	Hospital effluents	10	74
MTX	Composite sample (24h)	Effluent WWTP	8	12
	Grab sample	Influent WWTP	NA	24
	Grab sample	Effluent WWTP	NA	24
	Grab sample	River	NA	24
	Grab samples	Hospital effluent	65	31
	Grab samples	Drinking water	3	32
	Grab samples	Surface water	3	32
	Composite samples (24h)	Influent and effluent WWTP	10	35
	NA	Raw sewage of WWTP	NA	37
	Composite sample (24h)	Influent WWTP	3	41
	Composite sample (24h)	Effluent WWTP	3	41
	Grab sample	River	3	41
	Time-proportional sampling	Raw wastewater	8	51
	NA	Hospital effluent	3	52
	NA	Influent WW	3	52
	NA	Influent WWTP	4	56
	NA	Effluent WWTP	5	56
	Composite sample (24h)	Influent WW	48	57
	Composite sample (24h)	Effluent WW	48	57
	NA	Hospital effluent	3	60
	NA	Influent WWTP	3	60
	NA	Effluent WWTP	3	60
	NA	Surface water	6	60
	Composite sample (24h)	Influent WW	24	61
	Composite sample (24h)	Effluent WW	31	61
	Grab sample	Influent WW	3	66
	Grab sample	Effluent WW	3	66
	Grab sample	River	3	66
	Grab sample	Hospital WW	4	69
	Composite sample (24h)	Influent WWTP	4	69
	Composite sample (24h)	Effluent WWTP	2	69
	Composite sample	Hospital effluents	10	74
	Composite sample (24h)	Influent WWTP	1	75
	Composite sample (24h)	Effluent WWTP	1	75
	Grab sample	Coastal water	28	75
	Grab sample	Oceanic water	13	75
TAM	Grab sample	Effluent WWTP	3	7
	Grab sample	Surface water	2	7
	Grab sample	River	22	9
	Hourly interval	Effluent WWTP	45	10
	Hourly interval	River	30	10
	Composite sample (12h)	WTW samples	9	11
	Grab sample (*3)	Surface water	18	11
	Composite sample (24h)	Wastewater	37	17
	NA	River	NA	18
	NA	Effluent WW	NA	19
	NA	Tap water	NA	19

	NA	River	NA	19
	NA	Sea	NA	19
	NA	Influent WWTP	5	21
	NA	Effluent WWTP	5	21
	NA	River	10	21
	NA	Influent WWTP	3	25
	NA	Effluent WWTP	3	25
	NA	River	15	25
	Composite sampling (24h)	Influent WWTP	3	33
	Composite sampling (24h)	Effluent WWTP	2	33
	Composite sampling (2days)	Hospital effluent	63	33
	Grab samples	Drinking water	6	34
	Grab samples	River	12	34
	Grab samples	Effluent WWTP	6	34
	Composite samples (24h)	Effluent WWTP	20	36
	Grab samples	River	20	36
	NA	River	26	40
	Grab sample	River	12	43
	Grab sample	Effluent WWTP	6	43
	Composite sample (different depths)	River	30	44
	Composite sample (24h)	Hospital effluent	12	45
	Composite sample (24h)	Influent WWTP	4	45
	Composite sample (24h)	Effluent WWTP	4	45
	NA	River	NA	46
	Grab sample	River	24	47
	Composite sample (24h)	Effluent WWTP	6	48
	Composite sample	River	8	49
	Grab sample	Groundwater	31	50
	Grab sample	River	1	50
	Time-proportional sampling	Raw wastewater	8	51
	NA	Hospital effluent	3	52
	NA	Influent WW	3	52
	Grab sample	Influent WWTP	2	54
	Grab sample	Effluent WWTP	2	54
	Grab sample	River	549	58
	NA	Hospital effluent	3	60
	NA	Influent WWTP	3	60
	NA	Effluent WWTP	3	60
	NA	Surface water	6	60
	Composite sample (24h)	Influent WW	24	61
	Composite sample (24h)	Effluent WW	31	61
	Grab sample	River	40	64
	Grab sample	Effluent WWTP	20	64
	Grab sample	Influent WW	3	66
	Grab sample	Effluent WW	3	66
	Grab sample	River	3	66
	Grab sample	Hospital WW	4	69
	Composite sample (24h)	Influent WWTP	4	69
	Composite sample (24h)	Effluent WWTP	2	69

Table 3: Extraction and determination techniques of the four most frequently studied compounds

Compound	Extraction	Separation and detection	Concentration range in ng/L	Ref
CP	SPE: C18	GC-EI-MS	146	[1]
	SPE: C18	GC-EI-MS	6-4500	[2]
	SPE: PPL Bond-Elut	LC-ESI-MS-MS	n.d.	[4]
	SPE: LiChrolutEN	LC-ESI-MS-MS	n.d	[5]
	SPE: C18	LC-ESI-MS-MS	n.d.	[6]
	SPE: Lichrolut EN	LC-ESI-MS-MS	2.1-9	[12]
	SPE: Oasis	GC-EI-MS	<30-64.8	[13]
	SPE: macroporous polystyrene divinylbenzene adsorbent	LC-ESI-MS-MS	0.05-0.17	[16]
	SPE: macroporous polystyrene divinylbenzene adsorbent	GC-MS	2-11	[16]
	SPE: Oasis HLB	GC-EI-MS	45-65	[20]
	SPE: Oasis HLB	GC-EI-ITMS	n.d.	[20]
	SPE: MCX/MAX	LC-ESI-MS-MS	<25	[22]
	On-line SPE: Strata-X	LC-ESI-MS-MS	<5-9	[24]
	SPE: Strata-X	LC-ESI-MS-MS	<5	[27]
	SPE: Oasis HLB	LC-ESI-MS-MS	<100-<2000	[29]
	SPE: Oasis HLB	UPLC-QToF-MS-MS	33.3-114	[30]
	Bag-SPE: XAD-2	UPLC-QToF-MS-MS	114-150	[30]
	SPE: Oasis HLB	UPLC-ESI-MS-MS	4-2000	[31]
	On-line SPE: Strata-X	LC-ESI-MS-MS	<1	[32]
	SPE: Oasis HLB	LC-ESI-MS-MS	8.5-14.5	[35]
	SPE: Oasis HLB	LC-ESI-MS-MS	n.d.	[36]
	On-line SPE: Strata-X	LC-APPI-MS-MS	<11	[37]
	On-line SPE: Strata-X	LC-APCI-MS-MS	<7	[37]
	On-line SPE: Strata-X	LC-ESI-MS-MS	12	[37]
	SPE: Oasis MCX & Strata-X	UPLC-ESI-MS-MS	<26	[38]
	SPE: Strata-X & Florisil	LC-ESI-MS-MS	0.19-3.7	[39]
	SPE: Oasis HLB	LC-ESI-MS-MS	<1.7-<2.3	[41]
	SPE: Oasis HLB	LC-ESI-MS-MS	5730-13100	[42]
	On-line SPE: PLRP-s	LC-ESI-MS-MS	n.d.	[51]
	SPE: Oasis HLB	UPLC-ESI-MS-MS	25.5-200.7	[52]
	On-line SPE: Strata-X	LC-ESI-MS-MS	n.d.	[55]
	On-line SPE: Hypersil Gold PFP	LC-ESI-MS-MS	17-22	[56]
	SPE: Oasis HLB	LC-ESI-MS-MS	<2.13-<2.29	[57]
	SPE: Oasis HLB	LC-HESI-MS-MS	5-4720	[59]
	SPE: Oasis HLB	UPLC-ESI-MS-MS	7-43	[60]
	On-line SPE: PLRP-s	LC-ESI-MS-MS	2.4-100	[61]
	SPE: Oasis HLB	GC-EI-MS	15-22000	[62]
	SPE: Oasis MAX	LC-ESI-MS-MS	16-22	[64]
	SPE: Oasis HLB	GC-EI-MS	76-2686	[67]
	SPE: Oasis HLB	GC-EI-MS	6-22100	[69]
	SPE: Oasis HLB	LC-ESI-MS-MS	1-33.3	[70]
	SPE: Oasis HLB	LC-ESI-MS-MS	15-17	[72]
	MCPS	LC-ESI-MS-MS	19	[72]
	SPE: PS2/AC2, HLB/AC2, PLS-3/AC2	LC-ESI-MS-MS	n.d.	[73]
	On-line SPE: PLRP-s	LC-ESI-MS-MS	46-3000	[74]
IF	SPE: C18	GC-EI-MS	24	[1]
	SPE: RP-18	GC-EI-MS	<6-1914	[3]
	SPE: PPL Bond-Elut	LC-ESI-MS-MS	n.d.	[4]

	SPE: macroporous polystyrene divinylbenzene adsorbent	LC-ESI-MS-MS	<0.05-0.14	[16]
	SPE: macroporous polystyrene divinylbenzene adsorbent	GC-MS	1.4-<15	[16]
	SPE: Strata-X	LC-ESI-MS-MS	<25	[27]
	SPE: Oasis HLB	LC-ESI-MS-MS	<100-<2000	[29]
	SPE: Oasis HLB	UPLC-ESI-MS-MS	4-10647	[31]
	SPE: Oasis HLB	LC-ESI-MS-MS	9-16.4	[35]
	SPE: Oasis HLB	LC-ESI-MS-MS	n.d.	[36]
	SPE: Strata-X & Florisil	LC-ESI-MS-MS	n.d.	[39]
	SPE: Oasis HLB	LC-ESI-MS-MS	1.2-3.5	[41]
	On-line SPE: PLRP-s	LC-ESI-MS-MS	7.3-43.3	[51]
	SPE: Oasis HLB	UPLC-ESI-MS-MS	31.5-227.9	[52]
	On-line SPE: Strata-X	LC-ESI-MS-MS	n.d.	[55]
	On-line SPE: Hypersil Gold PFP	LC-ESI-MS-MS	<4	[56]
	SPE: Oasis HLB	LC-ESI-MS-MS	4.14-19.1	[57]
	SPE: Oasis HLB	LC-HESI-MS-MS	2690-86200	[59]
	SPE: Oasis HLB	UPLC-ESI-MS-MS	n.d.	[60]
	On-line SPE: PLRP-s	LC-ESI-MS-MS	2.2-27.9	[61]
	SPE: Oasis HLB	GC-EI-MS	48-6800	[62]
	SPE: Oasis HLB-MAX	cHPLC-ESI-MS-MS	<0.14-<0.31	[65]
	SPE: Oasis HLB	UPLC-ESI-MS-MS	<0.4-<1.53	[66]
	SPE: Oasis HLB	GC-EI-MS	26-47	[67]
	SPE: Oasis HLB-MAX	UHPSFC-ESI-MS-MS	<0.51-<0.58	[68]
	SPE: Oasis HLB	GC-EI-MS	<4.8-48	[69]
	SPE: Oasis HLB	LC-ESI-MS-MS	2.2-28	[70]
	SPE: Oasis HLB-MAX	cLC-ESI-MS-MS	<0.12-<0.28	[71]
	SPE: PS2/AC2, HLB/AC2, PLS-3/AC2	LC-ESI-MS-MS	n.d.	[73]
	On-line SPE: PLRP-s	LC-ESI-MS-MS	<0.2-4761	[74]
MTX	SPE: OASIS MCX	LC-ESI-MS-MS	12.6	[12]
	On-line SPE: Strata-X	LC-ESI-MS-MS	<4-59	[24]
	SPE: Oasis HLB	UPLC-ESI-MS-MS	4-4689	[31]
	On-line SPE: Strata-X	LC-ESI-MS-MS	<1-<2	[32]
	SPE: Oasis HLB	LC-ESI-MS-MS	1.6-18.1	[35]
	On-line SPE: Strata-X	LC-APPI-MS-MS	Not ionised	[37]
	On-line SPE: Strata-X	LC-APCI-MS-MS	Not ionised	[37]
	On-line SPE: Strata-X	LC-ESI-MS-MS	<11	[37]
	SPE: Oasis HLB	LC-ESI-MS-MS	<0.1	[41]
	On-line SPE: PLRP-s	LC-ESI-MS-MS	2.1-43.3	[51]
	SPE: Oasis HLB	UPLC-ESI-MS-MS	23	[52]
	On-line SPE: Hypersil Gold PFP	LC-ESI-MS-MS	13-60	[56]
	SPE: Oasis HLB	LC-ESI-MS-MS	7.30-55.8	[57]
	SPE: Oasis HLB	UPLC-ESI-MS-MS	5-23	[60]
	On-line SPE: PLRP-s	LC-ESI-MS-MS	2-19.4	[61]
	SPE: Oasis HLB	UPLC-ESI-MS-MS	<20.24-<29.83	[66]
	On-line SPE: PLRP-s	LC-ESI-MS-MS	8.3-3920	[69]
	On-line SPE: PLRP-s	LC-ESI-MS-MS	<0.1-4756	[74]
	SPE: Oasis HLB	LC-ESI-MS-MS	n.d.-3.5	[75]
TAM	SPE: Strata X	LC-ESI-MS-MS	<10	[7]
	SPE: Strata X	LC-ESI-MS-MS	<4-71	[9]
	SPE: Strata X	LC-ESI-MS-MS	<10-42	[10]
	SPE: Strata X	LC-ESI-MS-MS	27-740	[11]
	LLE	GC-EI-MS	1-4	[17]

	SPE: Oasis HLB	LC-ESI-MS-MS	<0.003	[18]
	SPE: Strata X	LC-ESI-MS-MS	<0.03	[19]
	SPE: Oasis HLB	LC-ESI-MS-MS	0.2-1.5	[21]
	SPE: Oasis HLB	LC-ESI-MS-MS	<1	[25]
	SPE: Oasis HLB	UPLC-ESI-MS-MS	0.2-8.2	[33]
	On-line SPE: HySphere Resin GP	LC-ESI-MS-MS	n.d.	[34]
	SPE: Oasis HLB	LC-ESI-MS-MS	n.d.	[36]
	SPE: Oasis HLB	UPLC-ESI-MS-MS	<0.05-19.57	[40]
	On-line SPE: HySphere Resin GP	LC-ESI-MS-MS	n.d.	[43]
	SPE: Oasis HLB	LC-ESI-MS-MS	0.2-1.8	[44]
	SPE: Oasis HLB	LC-ESI-MS-MS	<1-<2	[45]
	On-line TFC	LC-ESI-MS-MS	22.4-22.8	[46]
	On-line TFC	LC-ESI-MS-MS	12.4-26.8	[47]
	SPE: Oasis HLB	LC-HESI-MS-MS	13-24	[48]
	SPE: Oasis HLB	LC-ESI-MS-MS	0.01-115.04	[49]
	On-line SPE: HySphere Resin GP	LC-ESI-MS-MS	9.25-223	[50]
	On-line SPE: PLRP-s	LC-ESI-MS-MS	3.5-17.2	[51]
	SPE: Oasis HLB	UPLC-ESI-MS-MS	26.3-133.4	[52]
	SPE: Oasis HLB	LC-ESI-MS-MS	8-46	[54]
	SPE: Strata-X	LC-ESI-MS-MS	3.5-11.7	[58]
	SPE: Oasis HLB	UPLC-ESI-MS-MS	11-170	[60]
	On-line SPE: PLRP-s	LC-ESI-MS-MS	102-180.6	[61]
	SPE: Oasis MAX	LC-ESI-MS-MS	9-76	[64]
	SPE: Oasis HLB	UPLC-ESI-MS-MS	<3.5-<72.6	[66]
	On-line SPE: PLRP-s	LC-ESI-MS-MS	7.1-61	[69]

MCPS	Macroporous Ceramic Passive Sampler
PPL Bond Elut	Priority PolLutant Bond Elut
Oasis HLB	Hydrophilic-Lipophilic Balance
Oasis MCX	Mixed-mode, strong Cation-eXchange
Oasis MAX	Mixed-mode, strong Anion-eXchange
XAD-2	Polymer Resin
PLRP-s	Plymeric Reversed Phase
Hypersil Gold PFP	Hypersil Gold Perfluorinated Phenyl
PS2	Styrene-divinylbenzene copolymer
AC2	Activated Carbon
PLS-3	Nitrogen-Containing Methacrylate and SDB copolymer
RP-18	C18 (RP18, ODS, Octadecyl)
C-18	Non-Polar Sorbent (Silica)
HySphere Resin GP	General Phase (polydivinyl-benzene)
Online TFC	Online Turbulent Flow Chromatography
Strata-X	Polymeric SPE Sorbent
LLE	Liquid-liquid extraction

References

(1) Steger-Hartmann, T.; Kümmerer, K.; Schecker, J. Trace Analysis of the Antineoplastics Ifosfamide and Cyclophosphamide in Sewage Water by Two-Step Solid-Phase Extraction and Gas Chromatography-Mass Spectrometry. *J. Chromatogr. A* **1996**, *726* (1–2), 179–184.

(2) Steger-Hartmann, T.; Kummerer, K.; Hartmann, A. Biological Degradation of Cyclophosphamide and Its Occurrence in Sewage Water. *Ecotoxicol. Environ. Saf.* **1997**, *36* (2), 174–179.

(3) Kummerer, K.; Steger-Hartmann, T.; Meyer, M. Biodegradability of the Anti-Tumour Agent Ifosfamide and Its Occurrence in Hospital Effluents and Communal Sewage. *Water Res.* **1997**, *31* (11), 2705–2710.

(4) Sacher, F.; Lange, F. T.; Braus, H.-J.; Blankenhorn, I. Pharmaceuticals in Groundwaters Analytical Methods and Results of a Monitoring Program in Baden-Wurttemberg, Germany. *J. Chromatogr. A* **2001**, *938* (1–2), 199–210.

(5) Calamari, D.; Zuccato, E.; Castiglioni, S.; Bagnati, R.; Fanelli, R. Strategic Survey of Therapeutic Drugs in the Rivers Po and Lambro in Northern Italy. *Environ. Sci. Technol.* **2003**, *37* (7), 1241–1248.

(6) Kanda, R.; Griffin, P.; James, H. A.; Fothergill, J. Pharmaceutical and Personal Care Products in Sewage Treatment Works. *J. Environ. Monit.* **2003**, *5* (5), 823–830.

(7) Hilton, M. J.; Thomas, K. V. Determination of Selected Human Pharmaceutical Compounds in Effluent and Surface Water Samples by High-Performance Liquid Chromatography – Electrospray Tandem Mass Spectrometry. *J. Chromatogr. A* **2003**, *1015* (1–2), 129–141.

(8) Mahnik, S. N.; Rizovski, B.; Fuerhacker, M.; Mader, R. M. Determination of 5-Fluorouracil in Hospital Effluents. *Anal. Bioanal. Chem.* **2004**, *380* (1), 31–35.

(9) Thomas, K. V.; Hilton, M. J. The Occurrence of Selected Human Pharmaceutical Compounds in UK Estuaries. *Mar. Pollut. Bull.* **2004**, *49* (5–6), 436–444.

(10) Ashton, D.; Hilton, M.; Thomas, K. V. Investigating the Environmental Transport of Human Pharmaceuticals to Streams in the United Kingdom. *Sci. Total Environ.* **2004**, *333* (1–3), 167–184.

(11) Roberts, P. H.; Thomas, K. V. The Occurrence of Selected Pharmaceuticals in Wastewater Effluent and Surface Waters of the Lower Tyne Catchment. *Sci. Total Environ.* **2006**, *356* (1–3), 143–153.

(12) Castiglioni, S.; Bagnati, R.; Calamari, D.; Fanelli, R.; Zuccato, E. A Multiresidue Analytical Method Using Solid-Phase Extraction and High-Pressure Liquid Chromatography Tandem Mass Spectrometry to Measure Pharmaceuticals of Different Therapeutic Classes in Urban Wastewaters. *J. Chromatogr. A* **2005**, *1092* (2), 206–215.

(13) Moldovan, Z. Occurrences of Pharmaceutical and Personal Care Products as Micropollutants in Rivers from Romania. *Chemosphere* **2006**, *64* (11), 1808–1817.

(14) Mahnik, S. N.; Rizovski, B.; Fuerhacker, M.; Mader, R. M. Development of an Analytical Method for the Determination of Anthracyclines in Hospital Effluents. *Chemosphere* **2006**, *65* (8), 1419–1425.

(15) Mahnik, S. N.; Lenz, K.; Weissenbacher, N.; Mader, R. M.; Fuerhacker, M. Fate of 5-Fluorouracil, Doxorubicin, Epirubicin, and Daunorubicin in Hospital Wastewater and Their

Elimination by Activated Sludge and Treatment in a Membrane-Bio-Reactor System. *Chemosphere* **2007**, *66* (1), 30–37.

(16) Buerge, I. J.; Buser, H. R.; Poiger, T.; Müller, M. D. Occurrence and Fate of the Cytostatic Drugs Cyclophosphamide and Ifosfamide in Wastewater and Surface Waters. *Environ. Sci. Technol.* **2006**, *40* (23), 7242–7250.

(17) Tauxe-Wuersch, A.; De Alencastro, L. F.; Grandjean, D.; Tarradellas, J. Trace Determination of Tamoxifen and 5-Fluorouracil in Hospital and Urban Wastewaters. *Int. J. Environ. Anal. Chem.* **2006**, *86* (7), 473–485.

(18) Zhang, Z. L.; Zhou, J. L. Simultaneous Determination of Various Pharmaceutical Compounds in Water by Solid-Phase Extraction-Liquid Chromatography-Tandem Mass Spectrometry. *J. Chromatogr. A* **2007**, *1154* (1–2), 205–213.

(19) Nebot, C.; Gibb, S. W.; Boyd, K. G. Quantification of Human Pharmaceuticals in Water Samples by High Performance Liquid Chromatography-Tandem Mass Spectrometry. *Anal. Chim. Acta* **2007**, *598* (1), 87–94.

(20) Moldovan, Z.; Chira, R.; Alder, A. C. Environmental Exposure of Pharmaceuticals and Musk Fragrances in the Somes River before and after Upgrading the Municipal Wastewater Treatment Plant Cluj-Napoca , Romania. *Environ. Sci. Pollut. Res.* **2009**, *16* (Suppl 1), 46–54.

(21) Zhou, J. L.; Zhang, Z. L.; Banks, E.; Grover, D.; Jiang, J. Q. Pharmaceutical Residues in Wastewater Treatment Works Effluents and Their Impact on Receiving River Water. *J. Hazard. Mater.* **2009**, *166* (2–3), 655–661.

(22) Lavén, M.; Alsberg, T.; Yu, Y.; Adolfsson-Erici, M.; Sun, H. Serial Mixed-Mode Cation- and Anion-Exchange Solid-Phase Extraction for Separation of Basic, Neutral and Acidic Pharmaceuticals in Wastewater and Analysis by High-Performance Liquid Chromatography-Quadrupole Time-of-Flight Mass Spectrometry. *J. Chromatogr. A* **2009**, *1216* (1), 49–62.

(23) Kovalova, L.; McArdell, C. S.; Hollender, J. Challenge of High Polarity and Low Concentrations in Analysis of Cytostatics and Metabolites in Wastewater by Hydrophilic Interaction Chromatography/Tandem Mass Spectrometry. *J. Chromatogr. A* **2009**, *1216* (7), 1100–1108.

(24) Garcia-Ac, A.; Segura, P. A.; Gagnon, C.; Sauvé, S. Determination of Bezafibrate, Methotrexate, Cyclophosphamide, Orlistat and Enalapril in Waste and Surface Waters Using on-Line Solid-Phase Extraction Liquid Chromatography Coupled to Polarity-Switching Electrospray Tandem Mass Spectrometry. *J. Environ. Monit.* **2009**, *11* (4), 830–838.

(25) Gros, M.; Petrović, M.; Barceló, D. Tracing Pharmaceutical Residues of Different Therapeutic Classes in Environmental Waters by Using Liquid Chromatography/Quadrupole-Linear Ion Trap Mass Spectrometry and Automated Library Searching. *Anal. Chem.* **2009**, *81* (3), 898–912.

(26) Weissbrodt, D.; Kovalova, L.; Ort, C.; Pazhepurackel, V.; Moser, R.; Hollender, J.; Siegrist, H.; Mcardell, C. S. Mass Flows of X-Ray Contrast Media and Cytostatics in Hospital Wastewater. *Environ. Sci. Technol.* **2009**, *43* (13), 4810–4817.

(27) Busetti, F.; Linge, K. L.; Heitz, A. Analysis of Pharmaceuticals in Indirect Potable Reuse Systems Using Solid-Phase Extraction and Liquid Chromatography-Tandem Mass Spectrometry. *J. Chromatogr. A* **2009**, *1216* (31), 5807–5818.

(28) Mullot, J. U.; Karolak, S.; Fontova, A.; Huart, B.; Levi, Y. Development and Validation of a Sensitive and Selective Method Using GC/MS-MS for Quantification of 5-Fluorouracil in Hospital Wastewater. *Anal. Bioanal. Chem.* **2009**, *394* (8), 2203–2212.

(29) Ort, C.; Lawrence, M. G.; Reungoat, J.; Eaglesham, G.; Carter, S.; Keller, J. Determining the Fraction of Pharmaceutical Residues in Wastewater Originating from a Hospital. *Water Res.* **2010**, *44* (2), 605–615.

(30) Magnér, J. A.; Alsberg, T. E.; Broman, D. Bag-SPE-a Convenient Extraction Method for Screening of Pharmaceutical Residues in Influent and Effluent Water from Sewage Treatment Plants. *Anal. Bioanal. Chem.* **2009**, *395* (5), 1481–1489.

(31) Yin, J.; Shao, B.; Zhang, J.; Li, K. A Preliminary Study on the Occurrence of Cytostatic Drugs in Hospital Effluents in Beijing, China. *Bull. Environ. Contam. Toxicol.* **2010**, *84* (1), 39–45.

(32) Garcia-Ac, A.; Segura, P. A.; Viglino, L.; Fürtös, A.; Gagnon, C.; Prévost, M.; Sauvé, S. On-Line Solid-Phase Extraction of Large-Volume Injections Coupled to Liquid Chromatography-Tandem Mass Spectrometry for the Quantitation and Confirmation of 14 Selected Trace Organic Contaminants in Drinking and Surface Water. *J. Chromatogr. A* **2009**, *1216* (48), 8518–8527.

(33) Liu, X.; Zhang, J.; Yin, J.; Duan, H.; Wu, Y.; Shao, B. Analysis of Hormone Antagonists in Clinical and Municipal Wastewater by Isotopic Dilution Liquid Chromatography Tandem Mass Spectrometry. *Anal. Bioanal. Chem.* **2010**, *396* (8), 2977–2985.

(34) López-Serna, R.; Pérez, S.; Ginebreda, A.; Petrović, M.; Barceló, D. Fully Automated Determination of 74 Pharmaceuticals in Environmental and Waste Waters by Online Solid Phase Extraction-Liquid Chromatography- Electrospray-Tandem Mass Spectrometry. *Talanta* **2010**, *83* (2), 410–424.

(35) Yin, J.; Yang, Y.; Li, K.; Zhang, J.; Shao, B. Analysis of Anticancer Drugs in Sewage Water by Selective SPE and UPLC-ESI-MS-MS. *J. Chromatogr. Sci.* **2010**, *48* (10), 781–789.

(36) Gómez, M. J.; Gómez-Ramos, M. M.; Malato, O.; Mezcua, M.; Férnandez-Alba, A. R. Rapid Automated Screening, Identification and Quantification of Organic Micro-Contaminants and Their Main Transformation Products in Wastewater and River Waters Using Liquid Chromatography-Quadrupole-Time-of-Flight Mass Spectrometry with an Accurate-Mass . *J. Chromatogr. A* **2010**, *1217* (45), 7038–7054.

(37) Garcia-Ac, A.; Segura, P. A.; Viglino, L.; Gagnon, C.; Sauvé, S. Comparison of APPI, APCI and ESI for the LC-MS/MS Analysis of Bezafibrate, Cyclophosphamide, Enalapril, Methotrexate and Orlistat in Municipal Wastewater. *J. Mass Spectrom.* **2011**, *46* (4), 383–390.

(38) Nurmi, J.; Pellinen, J. Multiresidue Method for the Analysis of Emerging Contaminants in Wastewater by Ultra Performance Liquid Chromatography-Time-of-Flight Mass Spectrometry. *J. Chromatogr. A* **2011**, *1218* (38), 6712–6719.

(39) Llewellyn, N.; Lloyd, P.; Jürgens, M. D.; Johnson, A. C. Determination of Cyclophosphamide and Ifosfamide in Sewage Effluent by Stable Isotope-Dilution Liquid Chromatography-Tandem Mass Spectrometry. *J. Chromatogr. A* **2011**, *1218* (47), 8519–8528.

(40) López-Serna, R.; Petrović, M.; Barceló, D. Development of a Fast Instrumental Method for the Analysis of Pharmaceuticals in Environmental and Wastewaters Based on Ultra High Performance Liquid Chromatography (UHPLC)-Tandem Mass Spectrometry (MS/MS). *Chemosphere* **2011**, *85* (8), 1390–1399.

(41) Martín, J.; Camacho-Muñoz, D.; Santos, J. L.; Aparicio, I.; Alonso, E. Simultaneous Determination of a Selected Group of Cytostatic Drugs in Water Using High-Performance Liquid Chromatography-Triple-Quadrupole Mass Spectrometry. *J. Sep. Sci.* **2011**, *34* (22), 3166–3177.

(42) Gómez-Canela, C.; Cortés-Francisco, N.; Oliva, X.; Pujol, C.; Ventura, F.; Lacorte, S.; Caixach, J. Occurrence of Cyclophosphamide and Epirubicin in Wastewaters by Direct Injection Analysis-Liquid Chromatography-High-Resolution Mass Spectrometry. *Environ. Sci. Pollut. Res.* **2012**, *19* (8), 3210–3218.

(43) López-Serna, R.; Postigo, C.; Blanco, J.; Pérez, S.; Ginebreda, A.; de Alda, M. L.; Petrović, M.; Munné, A.; Barceló, D. Assessing the Effects of Tertiary Treated Wastewater Reuse on the Presence Emerging Contaminants in a Mediterranean River (Llobregat, NE Spain). *Environ. Sci. Pollut. Res.* **2012**, *19* (4), 1000–1012.

(44) Osorio, V.; Pérez, S.; Ginebreda, A.; Barceló, D. Pharmaceuticals on a Sewage Impacted Section of a Mediterranean River (Llobregat River, NE Spain) and Their Relationship with Hydrological Conditions. *Environ. Sci. Pollut. Res.* **2012**, *19* (4), 1013–1025.

(45) Verlicchi, P.; Al Aukidy, M.; Galletti, A.; Petrovic, M.; Barceló, D. Hospital Effluent: Investigation of the Concentrations and Distribution of Pharmaceuticals and Environmental Risk Assessment. *Sci. Total Environ.* **2012**, *430*, 109–118.

(46) López-Serna, R.; Petrović, M.; Barceló, D. Direct Analysis of Pharmaceuticals, Their Metabolites and Transformation Products in Environmental Waters Using on-Line TurboFlow^{TM} Chromatography-Liquid Chromatography-Tandem Mass Spectrometry. *J. Chromatogr. A* **2012**, *1252*, 115–129.

(47) López-Serna, R.; Petrović, M.; Barceló, D. Occurrence and Distribution of Multi-Class Pharmaceuticals and Their Active Metabolites and Transformation Products in the Ebro River Basin (NE Spain). *Sci. Total Environ.* **2012**, *440*, 280–289.

(48) Grabic, R.; Fick, J.; Lindberg, R. H.; Fedorova, G.; Tysklind, M. Multi-Residue Method for Trace Level Determination of Pharmaceuticals in Environmental Samples Using Liquid Chromatography Coupled to Triple Quadrupole Mass Spectrometry. *Talanta* **2012**, *100*, 183–195.

(49) Osorio, V.; Marcé, R.; Pérez, S.; Ginebreda, A.; Cortina, J. L.; Barceló, D. Occurrence and Modeling of Pharmaceuticals on a Sewage-Impacted Mediterranean River and Their Dynamics under Different Hydrological Conditions. *Sci. Total Environ.* **2012**, *440*, 3–13.

(50) López-Serna, R.; Jurado, A.; Vázquez-Suñé, E.; Carrera, J.; Petrović, M.; Barceló, D. Occurrence of 95 Pharmaceuticals and Transformation Products in Urban Groundwaters Underlying the Metropolis of Barcelona, Spain. *Environ. Pollut.* **2013**, *174*, 305–315.

(51) Negreira, N.; López de Alda, M.; Barceló, D. On-Line Solid Phase Extraction-Liquid Chromatography-Tandem Mass Spectrometry for the Determination of 17 Cytostatics and Metabolites in Waste, Surface and Ground Water Samples. *J. Chromatogr. A* **2013**, *1280*, 64–74.

(52) Ferrando-Climent, L.; Rodriguez-Mozaz, S.; Barceló, D. Development of a UPLC-MS/MS Method for the Determination of Ten Anticancer Drugs in Hospital and Urban Wastewaters, and Its Application for the Screening of Human Metabolites Assisted by Information-Dependent Acquisition Tool (IDA) in Sewage Samples. *Anal. Bioanal. Chem.* **2013**, *405* (18), 5937–5952.

(53) Kosjek, T.; Perko, S.; Žigon, D.; Heath, E. Fluorouracil in the Environment: Analysis, Occurrence, Degradation and Transformation. *J. Chromatogr. A* **2013**, *1290*, 62–72.

(54) Zhou, J.; Broodbank, N. Sediment-Water Interactions of Pharmaceutical Residues in the River Environment. *Water Res.* **2014**, *48* (1), 61–70.

(55) Idder, S.; Ley, L.; Mazellier, P.; Budzinski, H. Quantitative On-Line Preconcentration-Liquid Chromatography Coupled with Tandem Mass Spectrometry Method for the Determination of Pharmaceutical Compounds in Water. *Anal. Chim. Acta* **2013**, *805*, 107–115.

(56) Rabii, F. W.; Segura, P. A.; Fayad, P. B.; Sauvé, S. Determination of Six Chemotherapeutic Agents in Municipal Wastewater Using Online Solid-Phase Extraction Coupled to Liquid Chromatography-Tandem Mass Spectrometry. *Sci. Total Environ.* **2014**, *487* (1), 792–800.

(57) Martín, J.; Camacho-Munõz, D.; Santos, J. L.; Aparicio, I.; Alonso, E. Occurrence and Ecotoxicological Risk Assessment of 14 Cytostatic Drugs in Wastewater. *Water. Air. Soil Pollut.* **2014**, *225* (3), 1896.

(58) Iglesias, A.; Nebot, C.; Vázquez, B. I.; Coronel-Olivares, C.; Abuín, C. M. F.; Cepeda, A. Monitoring the Presence of 13 Active Compounds in Surface Water Collected from Rural Areas in Northwestern Spain. *Int. J. Environ. Res. Public Health* **2014**, *11* (5), 5251–5272.

(59) Gómez-Canela, C.; Ventura, F.; Caixach, J.; Lacorte, S. Occurrence of Cytostatic Compounds in Hospital Effluents and Wastewaters, Determined by Liquid Chromatography Coupled to High-Resolution Mass Spectrometry. *Anal. Bioanal. Chem.* **2014**, *406* (16), 3801–3814.

(60) Ferrando-Climent, L.; Rodriguez-Mozaz, S.; Barceló, D. Incidence of Anticancer Drugs in an Aquatic Urban System: From Hospital Effluents through Urban Wastewater to Natural Environment. *Environ. Pollut.* **2014**, *193*, 216–223.

(61) Negreira, N.; de Alda, M. L.; Barcelo, D. Cytostatic Drugs and Metabolites in Municipal and Hospital Wastewaters in Spain: Filtration, Occurrence, and Environmental Risk. *Sci. Total Environ.* **2014**, *497–498*, 68–77.

(62) Česen, M.; Kosjek, T.; Laimou-Geraniou, M.; Kompare, B.; Širok, B.; Lambropolou, D.; Heath, E. Occurrence of Cyclophosphamide and Ifosfamide in Aqueous Environment and Their Removal by Biological and Abiotic Wastewater Treatment Processes. *Sci. Total Environ.* **2015**, *527–528*, 465–473.

(63) Vidmar, J.; Martinčič, A.; Milačič, R.; Ščančar, J. Speciation of Cisplatin in Environmental Water Samples by Hydrophilic Interaction Liquid Chromatography Coupled to Inductively Coupled Plasma Mass Spectrometry. *Talanta* **2015**, *138*, 1–7.

(64) Azuma, T.; Ishiuchi, H.; Inoyama, T.; Teranishi, Y.; Yamaoka, M.; Sato, T.; Mino, Y. Occurrence and Fate of Selected Anticancer, Antimicrobial, and Psychotropic Pharmaceuticals in an Urban River in a Subcatchment of the Yodo River Basin, Japan. *Environ. Sci. Pollut. Res.* **2015**, *22* (23), 18676–18686.

(65) Camacho-Muñoz, D.; Kasprzyk-Hordern, B. Multi-Residue Enantiomeric Analysis of Human and Veterinary Pharmaceuticals and Their Metabolites in Environmental Samples by Chiral Liquid Chromatography Coupled with Tandem Mass Spectrometry Detection. *Anal. Bioanal. Chem.* **2015**, *407* (30), 9085–9104.

(66) Petrie, B.; Youdan, J.; Barden, R.; Kasprzyk-Hordern, B. Multi-Residue Analysis of 90 Emerging Contaminants in Liquid and Solid Environmental Matrices by Ultra-High-Performance Liquid Chromatography Tandem Mass Spectrometry. *J. Chromatogr. A* **2016**, *1431*, 64–78.

(67) Česen, M.; Kosjek, T.; Busetti, F.; Kompare, B.; Heath, E. Human Metabolites and Transformation Products of Cyclophosphamide and Ifosfamide: Analysis, Occurrence and Formation during Abiotic Treatments. *Environ. Sci. Pollut. Res.* **2016**, *23* (11), 11209–11223.

(68) Camacho-Muñoz, D.; Kasprzyk-Hordern, B.; Thomas, K. V. Enantioselective Simultaneous Analysis of Selected Pharmaceuticals in Environmental Samples by Ultrahigh Performance

Supercritical Fluid Based Chromatography Tandem Mass Spectrometry. *Anal. Chim. Acta* **2016**, *934*, 239–251.

(69) Isidori, M.; Lavorgna, M.; Russo, C.; Kundi, M.; Žegura, B.; Novak, M.; Filipič, M.; Mišïk, M.; Knasmueller, S.; de Alda, M. L.; et al. Chemical and Toxicological Characterisation of Anticancer Drugs in Hospital and Municipal Wastewaters from Slovenia and Spain. *Environ. Pollut.* **2016**, *219*, 275–287.

(70) Kot-Wasik, A.; Jakimska, A.; Śliwka-Kaszyńska, M. Occurrence and Seasonal Variations of 25 Pharmaceutical Residues in Wastewater and Drinking Water Treatment Plants. *Environ. Monit. Assess.* **2016**, *188* (12), 661.

(71) Camacho-Munoz, D.; Kasprzyk-Hordern, B. Simultaneous Enantiomeric Analysis of Pharmacologically Active Compounds in Environmental Samples by Chiral LC–MS/MS with a Macrocyclic Antibiotic Stationary Phase. *J. Mass Spectrom.* **2017**, *52* (2), 94–108.

(72) Franquet-Griell, H.; Pueyo, V.; Silva, J.; Orera, V. M.; Lacorte, S. Development of a Macroporous Ceramic Passive Sampler for the Monitoring of Cytostatic Drugs in Water. *Chemosphere* **2017**, *182*, 681–690.

(73) Chau, H. T. C.; Kadokami, K.; Ifuku, T.; Yoshida, Y. Development of a Comprehensive Screening Method for More than 300 Organic Chemicals in Water Samples Using a Combination of Solid-Phase Extraction and Liquid Chromatography-Time-of-Flight-Mass Spectrometry. *Environ. Sci. Pollut. Res.* **2017**, *24* (34), 26396–26409.

(74) Olalla, A.; Negreira, N.; López de Alda, M.; Barceló, D.; Valcárcel, Y. A Case Study to Identify Priority Cytostatic Contaminants in Hospital Effluents. *Chemosphere* **2018**, *190*, 417–430.

(75) Biel-Maeso, M.; Baena-Nogueras, R. M.; Corada-Fernández, C.; Lara-Martín, P. A. Occurrence, Distribution and Environmental Risk of Pharmaceutically Active Compounds (PhACs) in Coastal and Ocean Waters from the Gulf of Cadiz (SW Spain). *Sci. Total Environ.* **2018**, *612*, 649–659.
